# Budding Yeast Ubiquitin Ligases Rad5 and Rad18 Bind a Novel PCNA Surface, which Is Required for their Functions in DNA-Damage Tolerance

**DOI:** 10.7150/ijbs.124441

**Published:** 2026-02-26

**Authors:** Li Fan, Ying Du, Allison Novecosky, Yu Luo, Wei Xiao

**Affiliations:** 1 Department of Biochemistry, Microbiology and Immunology, University of Saskatchewan, Saskatoon, SK, Canada S7N 5E5.; 2 Beijing Key Laboratory of DNA Damage Responses and College of Life Sciences, Capital Normal University, Beijing, China 100048.

**Keywords:** *Saccharomyces cerevisiae*, PCNA, DNA-damage tolerance, Rad5, Rad18, HIRAN, RING

## Abstract

DNA-damage tolerance (DDT) is a highly regulated pathway to resume DNA synthesis in the presence of replication-blocking lesions. In budding yeast *Saccharomyces cerevisiae*, DDT is mediated by sequential ubiquitination and sumoylation of proliferating cell nuclear antigen (PCNA). Rad18 and Rad5 are two E3 ligases responsible for PCNA mono- and poly-ubiquitination, respectively, at its K164 residue. Although Rad18 and Rad5 have been reported to interact with PCNA, they do not contain known PCNA-binding domains like a PCNA interaction peptide (PIP) box. In this study, we performed extensive mapping by a yeast two-hybrid assay and delineated PCNA-binding regions within 40 residues in both Rad18 and Rad5; amino acid sequence alignment revealed that they share a previously uncharacterized sequence defined as a LxLF motif in this study. Interestingly, Rad5 and Rad18 interact with PCNA on same surface distinct from the PIP box-binding sites. Site-specific mutagenesis confirmed that this LxLF motif is required for the DDT functions of Rad18 and Rad5 possibly through affecting PCNA mono- and poly-ubiquitination, respectively. In addition, an adjacent HIRAN domain in Rad5, known to bind 3' DNA ends to drive replication fork reversal, is also required for the PCNA interaction and DDT functions, consistent with previous reports.

## Introduction

During eukaryotic DNA synthesis, replication forks can be stalled by base damage-induced DNA distortion, single-strand template DNA breaks or depletion of dNTPs, which collectively threaten the genome integrity and prevent the cell cycle progression. In addition to various DNA repair pathways that release these replication blocks, eukaryotes, from yeasts to human, possess a highly conserved DNA damage tolerance (DDT) mechanism to bypass those lesions with or without increased mutagenesis 1, 2. A unique feature of DDT is that it does not remove but rather tolerates replication-blocking lesions by resuming and completing genomic replication for cell survival 3-6. In the budding yeast *Saccharomyces cerevisiae*, DDT is coordinated through post-translational modifications (PTMs) of proliferating cell nuclear antigen (PCNA, encoded by *POL30* in budding yeast), an essential processive factor for the DNA synthesis, at a single K164 residue 7. Hence, when facing DNA damage that blocks replication, an E2-E3 ubiquitination complex Rad6-Rad18 mono-ubiquitinates PCNA-K164 and promotes translesion DNA synthesis (TLS) 7, 8. It turns out that Y-family TLS polymerases like Rev1 and Polη contain separate PCNA- and Ub-binding domains that can be recruited to the stalled replication fork by mono-ubiquitinated PCNA to replace replicative polymerase Polδ and resume local DNA synthesis. Mono-ubiquitinated PCNA can be further ubiquitinated by another E2-E3 complex Mms2-Ubc13-Rad5 that specifically promotes K63-linked polyUb chains, which leads to error-free lesion bypass or template switch by recruiting Shu (Csm2-Psy3-Shu1-Shu2) 9, Rad55-Rad57 10, 11 and Sgs1-Top3-Rmi1 complexes 12. In the absence of DNA-damage treatment, PCNA can be sumoylated by a SUMO E2-E3 complex Ubc9-Siz1 at the same K164 residue, which enhances physical interaction with a DNA helicase Srs2 to inhibit Rad51-ssDNA filament formation and prevent homologous recombination (HR), a process known as salvage HR 13-16.

PCNA is a homotrimer ring-shaped protein complex with pseudo sixfold symmetry and belongs to the DNA sliding clamp (β clamp) family, which are structurally and functionally conserved throughout living organisms 17, 18. Each budding yeast PCNA monomer has two independent and similar folded domains linked by a long, possibly flexible loop, called interdomain connector loop (IDCL) 17, 19. The three PCNA monomers arrange in a head-to-tail manner to form the inner positively charged α helices surface to interact with negatively charged DNA and the outer surface composed of β sheets 17, 19. The PCNA ring contains a front face including an IDCL region and a C-terminal region 17, and a back face 19, 20. Most PCNA interaction proteins contain a common PCNA-binding motif known as the PCNA interaction peptide (PIP) box, with a consensus sequence of Q-x-x-(h)-x-x-(a)-(a), where “x” represents any amino acids; “h” represents moderately hydrophobic side chains amino acids, including L, I, M; and “a” represents highly hydrophobic/aromatic side chains amino acids, including F and Y 21. In addition to the PIP box, a KA-box, in which a KA pair followed by 2-4 aliphatic hydrophobic residues mainly L 22, and an ABH2 PCNA-interaction motif (APIM) with a consensus sequence M-D-K/R-F-(L/V/I)2-K/R 23 were also found to be responsible for the PCNA interaction in several proteins involved in human DNA metabolism. To date, yeast proteins containing KA or APIM domains responsible for the PCNA interaction have not been reported.

Since Rad5 and Rad18 are PCNA E3 ubiquitin ligases, they must be responsible for the substrate recognition. Indeed, it has been reported that budding yeast Rad5 and Rad18 interact with PCNA in a yeast two-hybrid assay 7, but their PCNA-binding domains have not yet been identified. In this study, we demonstrated that budding yeast Rad5 and Rad18 do not contain a PIP box and bind PCNA on a common surface distinct from PIP-box proteins. We then systematically mapped PCNA-binding domains in both Rad5 and Rad18, which revealed that they share a consensus sequence designated as a putative LxLF motif. Indeed, this PCNA-binding motif is required for PCNA interaction both *in vitro* and *in vivo*, PCNA sequential ubiquitination and the DDT functions.

## Material and Methods

### Yeast strains and cell culture

Yeast strains used in this study are listed in Supplementary Table S1. A one-step gene deletion method 24 by using PCR-generated disruption cassettes 25 was used to generate gene deletion mutants. The target gene deletion mutants were confirmed by genomic PCR, followed by sequencing and phenotypic analysis. A LiAc/single-strand carrier DNA/PEG method 26 was used to transform the corresponding plasmid into yeast cells. *S. cerevisiae* cells were grown either in a rich yeast-extract peptone dextrose (YPD) broth (Sigma, Y1375) or YPD agar (Sigma, Y1500) medium. Strains requiring selection were cultured in synthetic defined (SD) medium with 6.706 g/L yeast nitrogen base without amino acid (USBiological, Y2025), 2% D-glucose (Sigma, G7021) and amino acid dropout mixes supplement (-His/-Leu/-Trp, Takara 630419; -Leu/-Trp, Takara 630417; -Ura, Takara 630416; -Leu, Takara 630414), add 2% Agar (Fisher, BP1423-500) when making plates. 5-fluoroorotic acid (5-FOA) plates were made as described before 27 to select for *ura3* auxotroph.

### Plasmid construction and site-specific mutagenesis

The indicated yeast genes or fragments were amplified by PCR using primers containing restriction enzyme cleavage sites as listed in Supplementary Table S2. The PCR product was cleaved by the corresponding restriction enzymes and cloned into vectors. In a yeast two-hybrid assay, pGBT9 and pGAD424 28 were used to produce Gal4_BD_ and Gal4_AD_ fusion proteins, respectively. In a GST pulldown assay, pET30a (source) and pGEX6 (source) were used to produce His_6_- and GST-tagged fusion proteins, respectively. For an *in vivo* co-IP assay, YCplac33 (YCp, *URA3*) and YEplac195 (YEp, *URA3*) 29 were used to create N-terminally 2×Flag tagged Rad5 or C-terminally 2×Flag tagged Rad18. For the DNA damage-sensitive serial dilution assay, *POL30* was cloned into YCplac111 (YCp, *LEU2*) 29. Positive clones were identified by colony PCR using primers as listed in Supplementary Table S2 and further confirmed by sequencing the entire insert.

Site-specific mutations were created by a modified Quick Change method 30, in which a pair of mutation primers with desired target amino acid substitution (Supplementary Table S2) were used to amplify the entire plasmid. The template DNA was digested by *Dpn*I (NEB, R0176S). The successful mutation was confirmed by sequencing the entire insert.

### Yeast two-hybrid assay

Plasmids encoding Gal4_AD_ and Gal4_BD_ fusion proteins were co-transformed into yeast strain PJ69-4a and individual colonies were picked from SD-Leu-Trp selective plates after 3-day incubation at 30 °C. At least two groups of individual colonies were cultured by SD-Leu-Trp liquid culture and spotted on an SD-Leu-Trp plate as control and on SD-Leu-Trp-His plates to test interactions. A histidine biosynthesis inhibitor 3-amino-1, 2, 4-triazole (3AT, Sigma A8056) was added to SD-Leu-Trp-His plates at certain concentrations to increase stringency. All plates were incubated for 2-6 days at 30 °C before photography with representative results shown in figures.

### Plasmid shuffling

A plasmid shuffling method 27 was used to introduce YCpL-Pol30 and its mutant alleles into yeast *pol30Δ* mutants as previously described 15. Briefly, yeast strain WXY939 (Table S1) contains a chromosomal *pol30Δ::HIS3* allele and its viability is maintained by plasmid pBL211 (YCp, *URA3*, *POL30*), a gift from Dr. P. Burgers (University of Washington, St Louis). To introduce *pol30* mutations, WXY939 cells were transformed with YCpL-Pol30 (YCp, *LEU2*, *POL30*) or its mutant derivatives. The resulting transformed cells were cultured in liquid YPD overnight, washed and then spread on a 5-FOA plate, followed by a 3-day incubation at 30 °C for 3 days. The 5-FOA resistant colonies were expected to lose plasmid pBL211 and hence can be used to assess the YCpL-Pol30 mutant phenotypes.

### Yeast cell survival serial dilution spot assay

A serial dilution assay was performed as previously described 31 to assess relative sensitivity of yeast mutants to different DNA-damaging agents. Briefly, overnight yeast cultures were adjusted to OD_600nm_ = 1.8 and used to make a set of 10-fold diluted samples, 4 or 6 µL was taken from each sample and spotted to freshly made YPD or SD selective agar plates, respectively, containing different concentrations of methyl methanesulfonate (MMS, Sigma 129925), 4-nitroquinoline N-oxide (4NQO, Sigma N8141) or hydroxyurea (HU, Sigma, H8627). The UV irradiation was by placing the spotted plate to a UV crosslinker (Stratagene SS-UV1800) at indicated doses. All plates were incubated for 2 days at 30 °C before photography. Only representative results were shown in the figures. In each set of experiment, cells were exposed to multiple doses/concentrations of DNA-damaging agents in duplicate, and only one representative set of plates was presented.

### GST pulldown assay

*E. coli* BL 21 was used to express His_6_ tagged Pol30, while Rosetta was used to express GST tagged Rad5, Rad18 and Rad30 fragments. After the 0.1 mM IPTG treatment and 16 °C incubation for 16-18 hrs, cells were harvested and resuspended in corresponding His_6_ lysis buffer (50 mM Tris-HCl, pH7.5, 150 mM NaCl, 30 mM imidazole, proteinase inhibitor) or GST lysis buffer (50 mM Tris-HCl, pH 8.0, 150 mM NaCl, 2 mM EDTA, 10 mM β-mercaptoethanol, proteinase inhibitor), followed by twice homogenization in a cell disruptor (Constant Cell Disruption Systems, CF1) at 25 PSI. His_6_-Pol30 was affinity purified by Ni sepharose (Cytiva, 17531801) and then eluted from the Ni sepharose by 5-fold bed volume of the His_6_ elution buffer (20 mM Tris-HCl, pH7.5, 200 mM NaCl, 500 mM imidazole, 20% glycerol). GST-Rad5, GST-Rad18 and GST-Rad30 were affinity purified by glutathione sepharose (Cytiva, GE17-0756-01) and stored in a GST stock buffer (50 mM Tris-HCl, pH 8.0, 150 mM NaCl, 2 mM EDTA, 10 mM β-mercaptoethanol, 20% glycerol). His_6_ and GST tagged proteins were mixing with equal molar and incubated overnight at 4 °C with gentle shaking. After thorough wash with GST lysis buffer, the glutathione sepharose was boiled 10 min with SDS-loading dye at 99 °C. Two identical protein gels were run and transformed onto PVDF membranes (Millipore, IPVH00010), one of which was incubated with anti-His_6_ (NEB, 12698, 1:1000) primary antibodies and another with anti-GST (Sigma, G7781-25UL, 1:1000) primary antibodies overnight with gentle shaking at 4 °C. An anti-rabbit (BioRad, 1706515, 1:3000) secondary antibody was used to incubate the above PVDF membranes at room temperature for 1 hr before imaging.

### *In vivo* co-IP assay

Overnight cultured yeast cells were harvested by centrifugation. 0.5 mL of 0.5 mm glass beads (BioSpec, 11079105) were added prior to dipping the 1.5 mL screw tube into liquid nitrogen. 150 µL lysis buffer (50 mM Tris-HCl pH 8.0, 150 mM NaCl, 1 mM MgCl_2_, 2 mM EDTA pH 8.0, 10% glycerol, 4 mM DTT, 10 mM β-mercaptoethanol, proteinase inhibitors) was added to every 7.5 OD_600nm_ cells followed by 2×2-min homogenization in a BioSpec mini-BeadBeater-12 (BioSpec Products, Bartlesville, OK, USA). After centrifugation for 10 min at top speed, the supernatant was collected as whole cell lysate and total protein concentrations were adjusted. A co-IP kit (Thermo Scientific, 26149) was used to immuno-precipitate Flag-tagged proteins by 15 µg anti-Flag M2 (Sigma, F1804) antibody coupled with the coupling resin after overnight incubation at 4 ^o^C. After extensive wash with the lysis buffer, samples were subjected to western blotting as described before by using anti-PCNA 5E6/2 (Abcam, Ab70472, 1:1000) and anti-Flag M2 (1:1000) monoclonal antibodies and the anti-mouse (Invitrogen, 31430, 1:3000) secondary antibody.

The western blot band intensity was measured by a BioRad Image Lab software (https://www.bio-rad.com/fr-ca/category/image-lab-software-suite?ID=5291f579-0715-48f4-b3de-766b92222582). Statistical analyses were performed by Microsoft Excel and the *P* value was calculated by the *t*-test from GraphPad Prism 9.0 (https://www.graphpad.com/updates/prism-900-release-notes).

### Western blotting to detect PCNA PTMs

Overnight cultures were used to inoculate 50-100 mL fresh YPD with 1:20 dilution and the incubation continued at 30 °C for 3-4 hr until OD_600nm_ = 0.35-0.4 to monitor PCNA sumoylation. To induce PCNA ubiquitinations, MMS at given concentrations was added and the incubation continued for the indicated period. 10 OD cells were pelleted, washed and resuspended in 0.5 mL sterile ddH_2_O, and then equal volume of 0.2 M NaOH was added followed by 15 min incubation at 24 °C. The supernatant was carefully removed, and the pellet was resuspended in 150 µL loading buffer containing 60 mM Tris-HCl, pH 6.8, 4% SDS, 0.01% bromophenol blue, 5% glycerol and 4% β-mercaptoethanol. The sample was boiled for 10 min before loading into 10% SDS-PAGE gel, and western blotting against the anti-PCNA monoclonal antibody (Abcam, Ab70472, 1:1000).

### Quantitative and statistical analyses

Western blot band intensity was quantified using Image Lab software (https://www.bio-rad.com/fr-ca/category/image-lab-software-suite?ID=5291f579-0715-48f4-b3de-766b92222582). The volume tools feature was used to measure band intensity in an unbiased manner. Briefly, rectangular volume boxes were drawn around each band, and background subtraction was performed using the local background subtraction method to minimize noise. Band intensities of Pol30 PTMs were first normalized by the corresponding Pol30 loading control (short exposure), then to Pol30-K164R (negative control, 0) and Pol30 (positive control, 1) and expressed as relative intensity values.

Data were analyzed from at least three independent experiments, and statistical analyses were performed by GraphPad Prism 9.0 (https://www.graphpad.com/updates/prism-900-release-notes) and the two-tailed *P* value was calculated by the unpaired *t*-test from GraphPad Prism 9.0.

### Protein structural and bioinformatics analysis

The *S. cerevisiae* PCNA structure 32 was obtained from the Protein Data Bank (https://www.rcsb.org/structure/1PLQ) and displayed by using Molscript 33. Multiple alignments were performed and presented by BioEdit 7.2 (https://bioedit.software.informer.com/7.2/).

Both ColabFold 34, which is based on AlphaFold 2 35, and AlphaFold 3 36 were used to build five models each for PCNA-Rad5, PCNA trimer-Rad5, PCNA-Rad18 and PCNA trimer-Rad18 complexes. All PCNA trimers were modeled as closed rings as expected. Consistent PCNA-Rad5 interface and PCNA-Rad18 interface were constructed regardless of whether PCNA monomer or trimer was modeled.

## Results

### Rad5 and Rad18 share a PCNA binding surface distinct from PIP-box proteins

**Figure 1:**
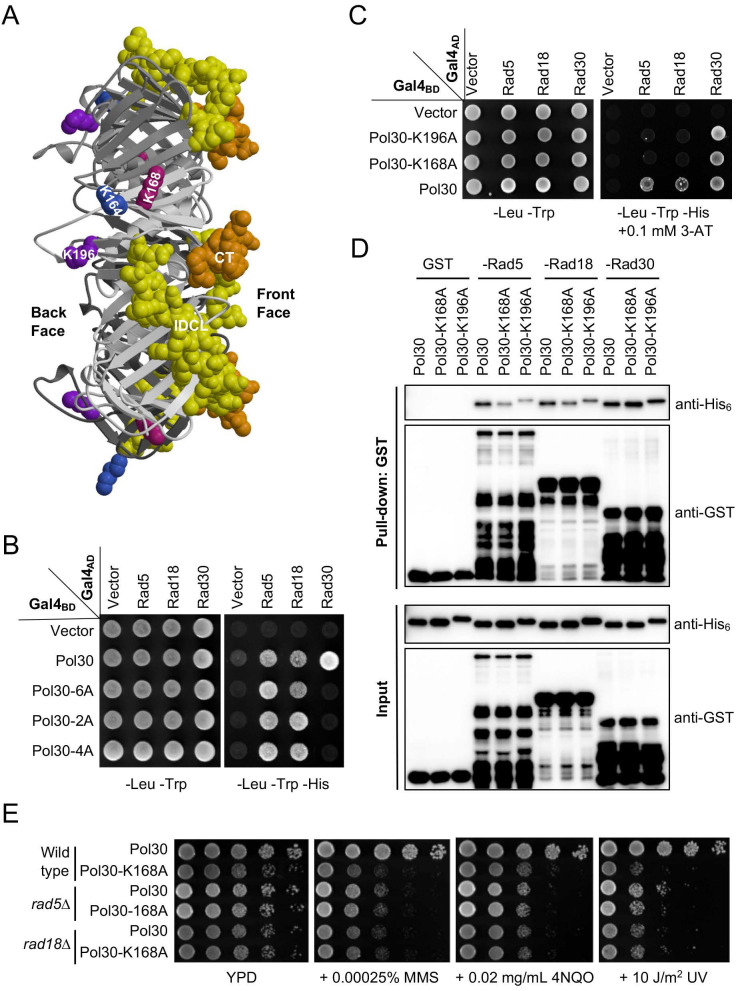
**Rad5 and Rad18 interact with PCNA differently from a PIP box protein Rad30**. (**A**) Side view of the budding yeast PCNA/Pol30 structure from the Protein Data Bank 1PLQ. The PCNA trimer is plotted with various shades of gray. The interdomain connector loop (IDCL) and C-terminus (CT) involved in PIP-box binding are highlighted in yellow and goldenrod, respectively. K164, K168 and K196 residues are indicated by royal blue, violet-red and purple, respectively. (**B**, **C**) Physical interactions between Pol30 point mutations and Rad5/Rad18/Rad30 by a Y2H assay. 2A refers to L126A, I128A located in IDCL; 4A refers to 252-255AAAA located at the C-terminus, while 6A combines both 2A and 4A mutations. The co-transformants were printed onto control SD-Leu-Trp plates and SD-Leu-Trp-His plates with or without different concentrations of 3-AT, and the plates were incubated at 30 ^o^C for 2-3 days before photography. At least two sets of independent transformants were tested with comparable results, and only one set of representative images is presented. (**D**) Physical interactions between Rad5/Rad18/Rad30 and Pol30 point mutations by a GST affinity pulldown assay. GST-fused Rad5-N431, Rad18-N160 and Rad30-(515-632) were produced and purified from bacterial cells, mixed with purified His_6_-Pol30 and a GST affinity pulldown followed by western blotting using antibodies against His_6_ and GST tags. (**E**) Relative sensitivity of *pol30*-*K168A* and *rad5∆/rad18*∆ mutants to DNA-damaging agents by a serial dilution assay. The *pol30* mutants were created by plasmid shuffling in either wild type or *rad5∆/rad18*∆ cells. Cells were cultured overnight, density adjusted, serially diluted by tenfold and spotted onto YPD plates containing indicated concentrations of chemicals. For the UV irradiation, the plates were exposed to UV in a UV crosslinker at the given dose. The plates were incubated at 30 ^o^C for two days before photography. At least two sets of independent cultures and multiple doses of DNA damage were employed and only selected representative images are shown.

Previous studies have mapped a PCNA-binding region to the Rad5 N-terminal 393 37 or 431 38 amino acids, while mapping of a PCNA-binding region in Rad18 has not been previously reported. Search for the above putative PCNA-binding regions did not reveal known PCNA interaction motifs including the PIP box. Budding yeast PIP-box proteins like Cdc9 39 interact with an IDCL motif (aa 121-132) and residues near the C-terminal region (CT, aa 251-258); both are located in the front face of PCNA (Figure 1A) 19, 20. To ask whether Rad5 and Rad18 contain a PIP box responsible for the PCNA binding, we examined PCNA mutations that specifically disrupt PIP-box binding to see if they affect physical interaction with Rad5/Rad18. We previously created three mutations *pol30-2A* (Pol30-L126A, I128A in conserved IDCL residues), *pol30-4A* (Pol30-PKFN252-255AAAA at the C-terminus) and *pol30-6A* (*pol30-2A+pol30-4A*) that specifically affect interaction with PIP-box proteins 15. In a Y2H assay, Pol30 can interact with a PIP-box protein Rad30 40. As anticipated, all three mutated Pol30s failed to bind Rad30; however, they do not affect physical interaction with Rad5 or Rad18 (Figure 1B), confirming that Rad5 and Rad18 do not share a binding surface on PCNA with PIP box proteins and hence do not contain a typical PIP box.

**Figure 2:**
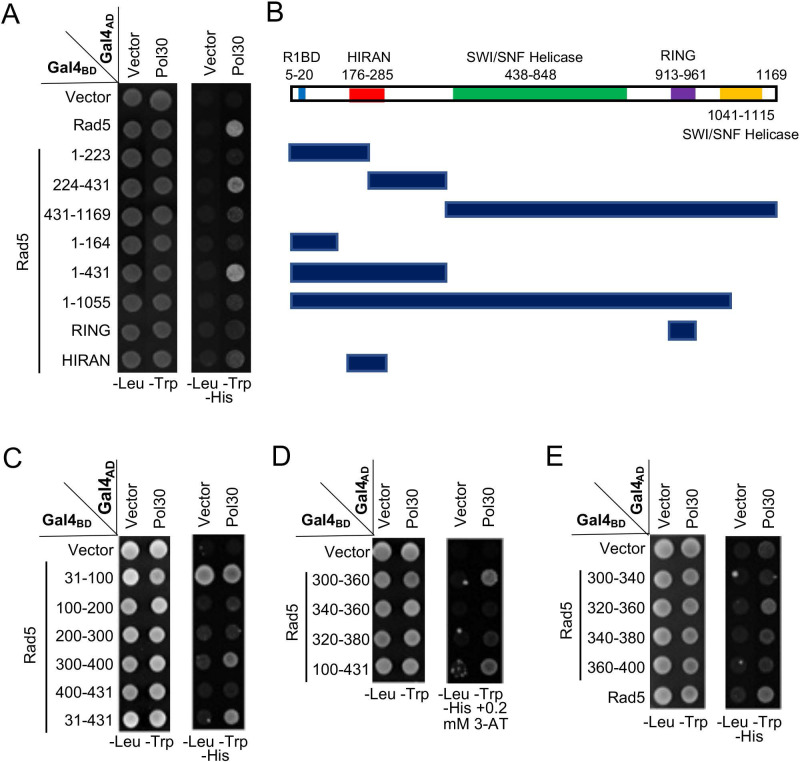
** Mapping the PCNA-binding region on Rad5 by a Y2H assay**. (**A**) Various Rad5 truncations based on available restriction sites were made and used to map the PCNA interaction region. (**B**) Schematic illustration of Rad5 truncations used in (A). R1BD: Rev1 binding domain. (**C**-**E**) Fine mapping of the PCNA-binding domain within Rad5-N431. Several arbitrary Rad5 truncations were made and used to map the PCNA interaction domain. Experimental conditions were as described in Figure 1B. At least two sets of independent transformants were tested with comparable results, and only one set of representative images is presented.

It was previously reported that a Pol30-K196A substitution affected its interaction with Rad18 41, while a Pol30-K168A substitution affected its interaction with Rad5 42. To test a hypothesis that both Rad5 and Rad18 bind to the same surface of PCNA, we performed a Y2H assay. As shown in Figure 1C, Pol30-K168A and -196A abolished interaction with full-length Rad5 and Rad18, while their interactions with Rad30 were not affected. Since Rad5 also interacts with Rad18 43, one cannot rule out a possibility that some of the above results are due to indirect interaction in yeast cells. To ask whether Rad5 and Rad18 can independently bind PCNA, we performed an affinity pulldown assay, in which truncated Rad5-N431 38 and Rad18-N160 (see Figure 2) known to interact with PCNA were used, as bacterial expression of soluble full-length Rad5 and Rad18 fusion proteins was a challenge. Indeed, purified GST fused Rad5 and Rad18 fragments were able to pulldown His_6_-tagged PCNA, but their interaction with Pol30-K168A and -K196A was significantly reduced. In contrast, the above two PCNA mutations did not affect interaction with a Rad30 fragment containing the PIP-box (Figure 1D). Furthermore, *pol30-K168A* and *pol30-K196A* cells are sensitive to a variety of DNA-damaging agents that cause common consequences of blocking replication, and the level of sensitivity is comparable to *pol30-K164R* (Supplementary Figure S1), indicating that their reduced interaction with Rad5 and Rad18 correlates with lack of PCNA PTMs. To further address whether *pol30-K168A/K196A* and *rad5/rad18* mutations affect DDT via the same mechanism, we created *rad5∆ pol30-K168A* and *rad18∆ pol30-K168A* double mutants and found that they were as sensitive to selected DNA-damaging agents as corresponding *rad5*∆ and *rad18*∆ single mutants (Figure 1E), confirming an epistatic relationship between *rad5/rad18* and *pol30-K168A*. Taken together, we conclude that Rad5 and Rad18 bind to the same surface on PCNA represented by K168 and K196 that is distinct from PIP-box proteins.

### Mapping PCNA-binding regions in Rad5 and Rad18

Rad5 is a large protein with several domains of known functions. During the mapping of the Rev1-binding domain 38, we have made a few truncations in pGBT-Rad5 by using available restriction sites within the *RAD5* coding region. These constructs were used for a Y2H assay against pGAD-Pol30, which mapped the PCNA-binding domain within N-terminal 431 residues and then aa 224-431 (Figure 2A, B). Subsequently, a systematic approach narrowed the PCNA-binding region to residues 300-400 (Figure 2C), 300-360 (Figure 2D) and finally to aa 320-360, although aa 340-380 also appeared to have a residual signal (Figure 2E).

**Figure 3:**
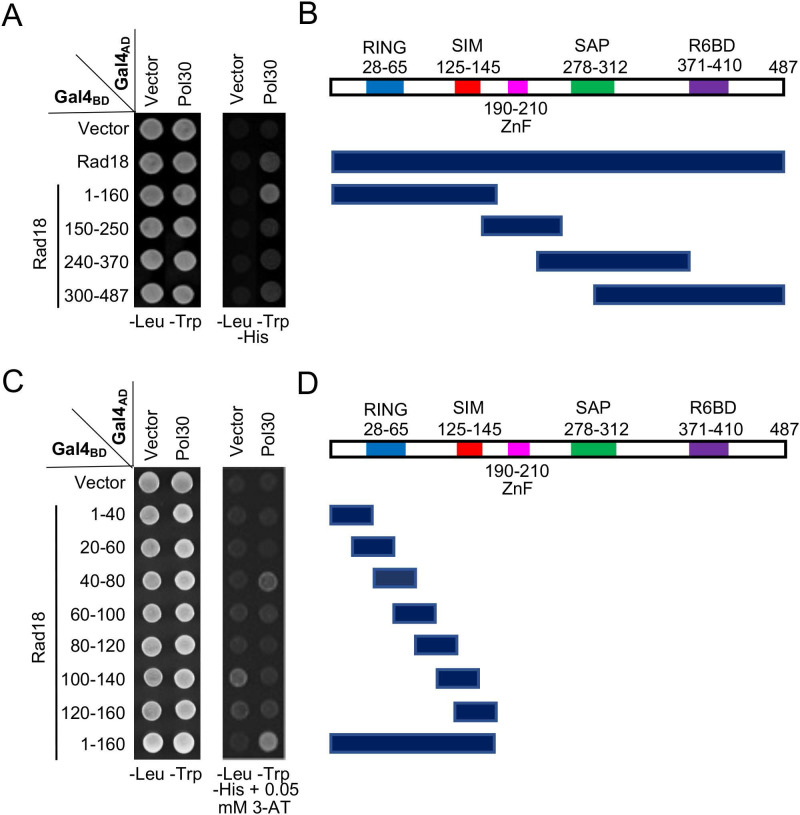
** Mapping the PCNA-binding region on Rad18 by a Y2H assay**. (**A**) Several Rad18 truncations were made based on known functional domains and used to map the PCNA interaction region. (**B**) Schematic illustration of Rad18 truncations used in (A). R6BD: Rad6 binding domain; ZnF: Zinc-finger domain. (**C,D**) Fine mapping of the PCNA-binding domain within Rad18-N160. Several arbitrary Rad18 truncations were made and used to map the PCNA interaction domain. Experimental conditions were as described in Figure 1B. At least two sets of independent transformants were tested with comparable results, and only one set of representative images is presented.

Rad18 is known to interact with PCNA in a Y2H assay 7; however, its interaction domain has not been mapped. In this study, Rad18 was truncated into several pieces based on its known functional domains, cloned into pGBT9, and the PCNA interaction region was mapped to the N-terminal 160 aa (Figure 3A, B). Subsequently, a set of 40-aa overlapping truncations was made within pGBT-Rad18-N160 and tested against pGAD-Pol30 in a Y2H assay, which mapped the PCNA interaction domain to residues 40-80 (Figure 3C, D).

### Rad5 and Rad18 require an LxLF consensus sequence to bind PCNA

**Figure 4:**
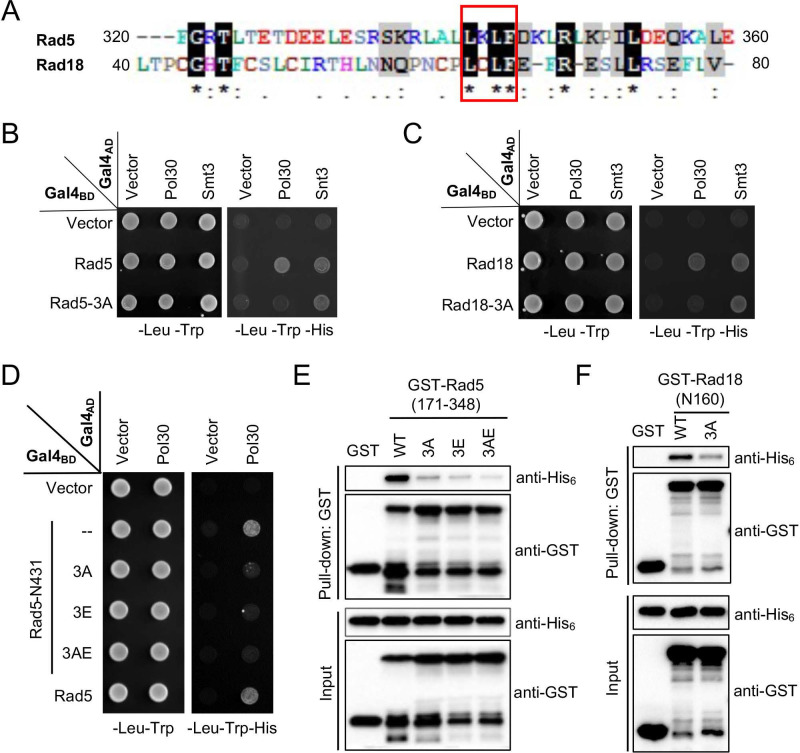
**Identification and characterization of an LxLF motif from Rad5 and Rad18**. (**A**) Sequence alignment of the mapped minimum PCNA-binding regions in Rad5 and Rad18. The sequence alignment was performed by BioEdit 7.2. Residues highlighted in black are identical sequences and those in grey are conserved residues. (**B**,** C**) Physical interactions between Rad5/Rad18 and Pol30 by a Y2H assay. The 3A mutation indicates LxLF-to-AxAA amino acid substitution. Rad5 and Rad18 interactions with Smt3 served as positive controls, respectively. (**D**) Effects of 3A and 3E amino acid substitutions on the physical interaction between Rad5-N431 and Pol30 by a Y2H assay. The 3E mutation contains R187E, R229E, R241E triple substitutions; 3AE contains both 3A and 3E mutations. (**E**, **F)** Assess physical interactions between Rad5 (177-348)/Rad18-N160 and Pol30 by a GST affinity pulldown assay. Experimental conditions were as described in Figure 1B and 1D. For the Y2H assay, at least two sets of independent transformants were tested with comparable results, and only one set of representative images is presented.

Since PCNA-binding domains on both Rad5 and Rad18 have been narrowed to a 40-aa region (Figures 2 and 3) and that the two proteins have been proposed to bind the same surface on PCNA (Figure 1), we aligned the two 40-aa sequences and identified a few conserved residues, among which clustered amino acids Leu, Leu and Phe caught our attention (Figure 4A) since they may form a hydrophobic pocket to play a role in the interaction with PCNA. We herein designated this sequence as an LxLF motif, in which “x” could potentially be any amino acid.

To ask whether the LxLF motif in Rad5 and Rad18 is required for the interaction with PCNA, a triple amino acid substitution was made in pGBT-Rad5 and pGBT-Rad18 to replace conserved Leu, Leu and Phe residues with Ala, which was designated as 3A. In a Y2H assay, the 3A mutation in both Rad5 (L341A, L343A and F344A; Figure 4B) and Rad18 (L64A, L66A and F67A; Figure 4C) abolished interaction with PCNA, consistent with our prediction. To rule out a possibility that the 3A mutation destabilized the truncated Gal4_BD_ fusion proteins, we took advantage that both Rad5 (our unpublished observation) and Rad18 44 contains a Smt3/SUMO-binding domain, and demonstrated that their interactions with Smt3 were not affected by the 3A mutation in the same set of experiments (Figure 4B, C). These experiments support a notion that the newly defined LxLF-containing domain is both required and sufficient for the Rad5 and Rad18 interaction with PCNA. We also examined the effects of individual amino acid substitutions of the LxLF motif on the PCNA binding. For Rad5-N431, L341A does not appear to affect PCNA interaction, whereas L343A and F344A appear to destabilize the truncated fusion protein, as the control interaction with Rev1 is severely affected (Supplementary Figure S2A). Meanwhile, all three individual amino acid substitutions (L64A, L66A and F67A) in Rad18-N160 caused false-positive signals in the Y2H assay (Figure S2B). These observations prevent us from further addressing relative contributions of each critical residue within the LxLF motif by this assay.

Interestingly, the Rad5 HIRAN domain, previously known to bind 3' single-strand DNA ends and drive replication fork reversal 45, 46, has also been implicated to play a role in the interaction with PCNA 47, 48. Specifically, a triple Arg-to-Glu (R187E, R229E and R241E or 3RE, designated as 3E in this study) mutation within the Rad5 HIRAN domain has been shown to reduce interaction with PCNA 48. Hence, we evaluated effects of the 3E mutation together with the 3A mutation in Rad5-N431 on their interaction with PCNA in a Y2H assay and found that both mutations abolished the interaction under our experimental conditions (Figure 4D).

To further address relative roles of Rad5 HIRAN and LxLF motifs in the PCNA interaction, we performed an affinity pulldown assay in which purified GST-tagged Rad5 aa 171-348 truncation and its mutant derivatives were incubated with His_6_-tagged Pol30 followed by GST affinity pulldown and extensive washing. As shown in Figure 4E, both truncated Rad5-3A and 3E mutations significantly reduced binding to PCNA, and the combined 3AE mutation appeared to further reduce PCNA binding, indicating that both HIRAN and LxLF-containing domains in Rad5 contribute to PCNA binding. We also performed an affinity pulldown assay for Rad18-N160 and found that its 3A mutation severely reduced interaction with PCNA (Figure 4F). The above results collectively demonstrate that an LxLF motif is required for the Rad5 and Rad18 interaction with PCNA.

### The LxLF motif in Rad5 and Rad18 is required for PCNA interaction *in vivo*

**Figure 5:**
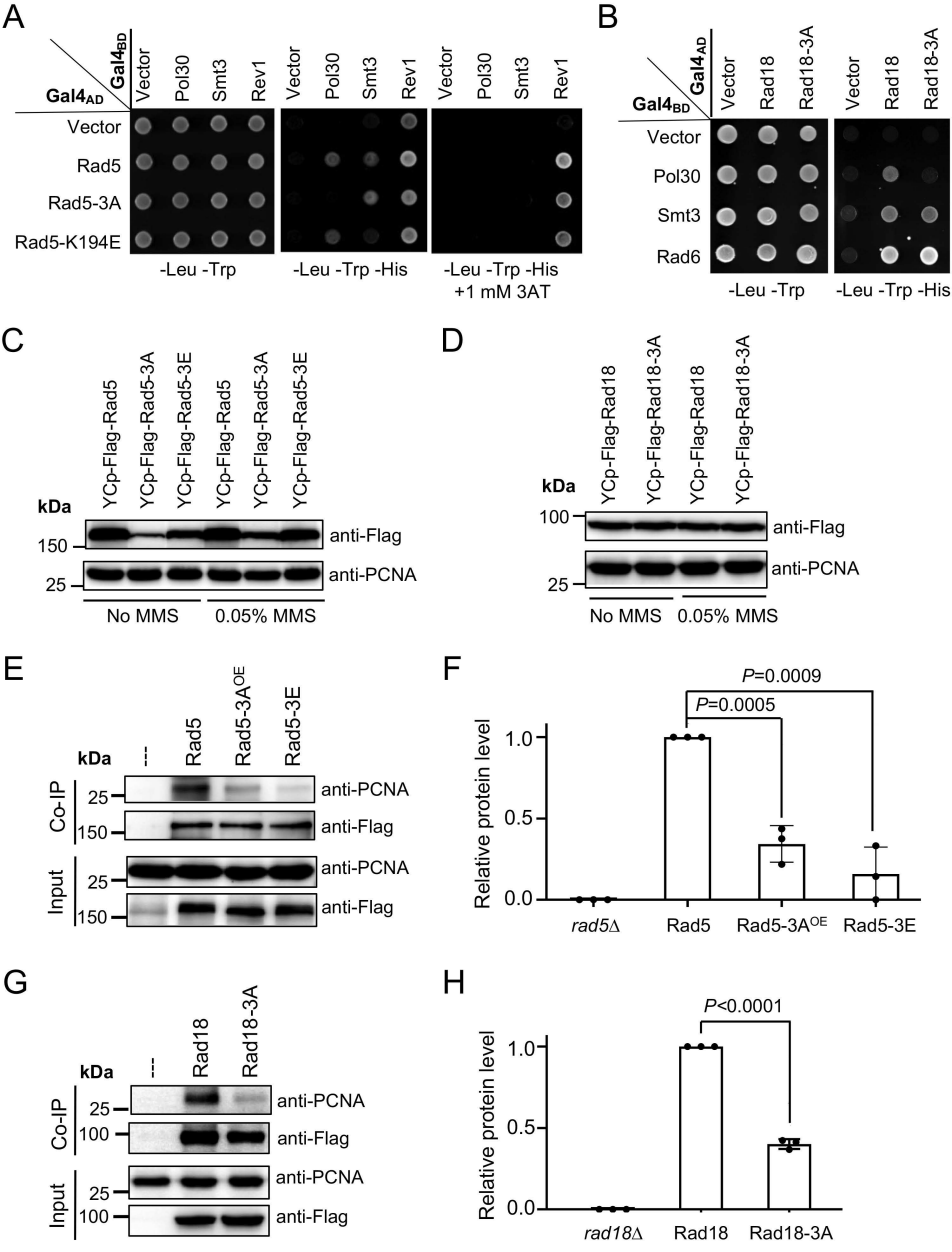
**Physical interactions of full-length Rad5/Rad18 and their derivatives with Pol30 *in vivo*.** (**A**) Physical interaction of full-length Rad5 and its mutant derivatives with Pol30 in a Y2H assay. (**B**) Physical interaction of full-length Rad18 and Rad18-3A with Pol30 in a Y2H assay. Rev1 and Smt3 serve as positive controls for Rad5, and Rad6 and Smt3 serve as positive controls for Rad18. Experimental conditions were as described in Figure 1B. (**C**) Relative Flag-Rad5 protein levels from *rad5*∆ cells transformed with YCp-based plasmids with or without 0.05% MMS treatment. (**D**) Relative Rad18-Flag protein levels from *rad18*∆ cells transformed with YCp-based plasmids with or without 0.05% MMS treatment. (**C**, **D**) Same amounts of cell pellets were collected. Equal amounts of whole-cell extracts (WCEs) were loaded into the protein gel, in which PCNA served as an internal reference. (**E**) Physical interaction between Rad5 and Pol30 in an *in vivo* co-IP assay. Flag-Rad5 and its mutant derivatives were immunoprecipitated by an anti-Flag antibody from WCEs and analyzed by WB against anti-Flag and anti-PCNA antibodies. Rad5-3A^OE^ indicates that the rad5∆ cells were transformed with a YEp-Flag-rad5-3A plasmid. (**F**) Quantitative analysis of data as shown in (E). (**G**) Physical interaction between Rad18 and Pol30 in an *in vivo* co-IP assay. Experimental conditions were as described in (E) except that Rad18-Flag and its mutant derivative were immunoprecipitated. (**H**) Quantitative analysis of data as shown in (G). For each experiment, at least three independent western blots were obtained and used for quantitative analysis. The co-IP anti-PCNA relative protein level was calculated by using co-imunoprecipitated anti-PCNA dually normalized against immunoprecipitated anti-Flag and input anti-PCNA. The results are presented as average relative protein levels ± standard deviations shown as error bars (n=3). Two-tailed *P* values from the unpaired *t*-test are shown.

To ask whether the LxLF motif is also required for PCNA binding *in vivo*, we performed two sets of experiments. Firstly, full-length Rad5 (Figure 5A) and Rad18 (Figure 5B) and their 3A derivatives were cloned into pGAD424 to produce Gal4_AD_ fusion proteins against Gal4_BD_-Pol30 in a Y2H assay, in which Rad5 and Rad18 interacted with PCNA as previously shown (Figure 1), while their 3A derivatives failed to bind PCNA under same experimental conditions (Figure 5A, B). To rule out a possibility that the lack of PCNA interaction by 3A mutations was due to reduced protein stability, we included Rev1 and Smt3 as internal controls for Rad5, and Rad6 and Smt3 as internal controls for Rad18. Since Rad5-3A (Figure 5A) and Rad18-3A (Figure 5B) mutations did not affect Rev1, Rad6 and Smt3 binding, we conclude that Rad5-3A and Rad18-3A specifically compromise interaction with PCNA but do not affect other interaction partners.

Secondly, a co-IP assay was conducted to assess Rad5-PCNA and Rad18-PCNA interactions *in vivo*. The ORFs of *RAD5* and *RAD18* along with their native promoter and terminator sequences and a 2xFlag tag were cloned into a single-copy plasmid YCplac33 29 to form YCpU-*Flag-Rad5* and YCpU-*Rad18-Flag*, respectively. Desired amino acid substitutions were then made by site-specific mutagenesis. These plasmids were used to transform corresponding *rad5* and *rad18* null mutant strains, and relative Rad5 and Rad18 protein levels were assessed by western blotting. As seen in Supplementary Figure S3A, and quantified in Figure S3B and C, *rad5-3A* and *rad5-3E* mutations substantially reduced cellular Rad5 protein levels under both untreated (Figure S3B) and MMS treated (Figure S3C) conditions. Interestingly, 0.05% MMS treatment led to a moderate increase in Rad5 mutant protein levels relative to the wild-type Rad5 (Figures 5C and S3D). To compensate such a defect, we cloned *Flag*-*RAD5* and its mutant derivatives into the corresponding multicopy plasmid vector YEplac195 29. Western blotting analyses indicate that YEp-*rad5-3A* transformed *rad5*∆ cells produced no less Flag-Rad5 protein than the YCp-*RAD5* transformed cells (Figure S3A-C), which was used for subsequent studies. On the other hand, Rad18-3A does not affect the Rad18 protein level regardless of MMS treatment (Figures 5D and S3E). The co-IP experimental results demonstrate that both Rad5-3A and Rad5-3E substitutions significantly reduced PCNA binding (Figures 5E, F and S4A, B), and that the Rad18-3A substitution also significantly reduced binding to PCNA (Figure 5G, H and Figure S4C, D).

The Rad5 HIRAN domain is required to bind structured DNA 45. To ask whether this DNA-binding activity is required for the PCNA interaction, we made a *rad5-K194E* mutation known to abolish the 3' end DNA-binding activity 45, 46. Interestingly, the *rad5-K194E* mutation did not affect PCNA and Rev1 binding but affected the Smt3 interaction (Figure 5A). Since the LxLF motif in Rad18 is embedded in the RING domain, we also asked whether its RING mutations affect PCNA binding. As shown in Fig. S2C, Rad18-C28S and Rad18-C65S substitutions abolished interactions with both Pol30 and Smt3 and reduced interaction with Rad6 in a Y2H assay, suggesting that disruption of the Rad18 RING domain has global effects on the protein structure and stability.

### The LxLF motif is required for PCNA ubiquitination

**Figure 6:**
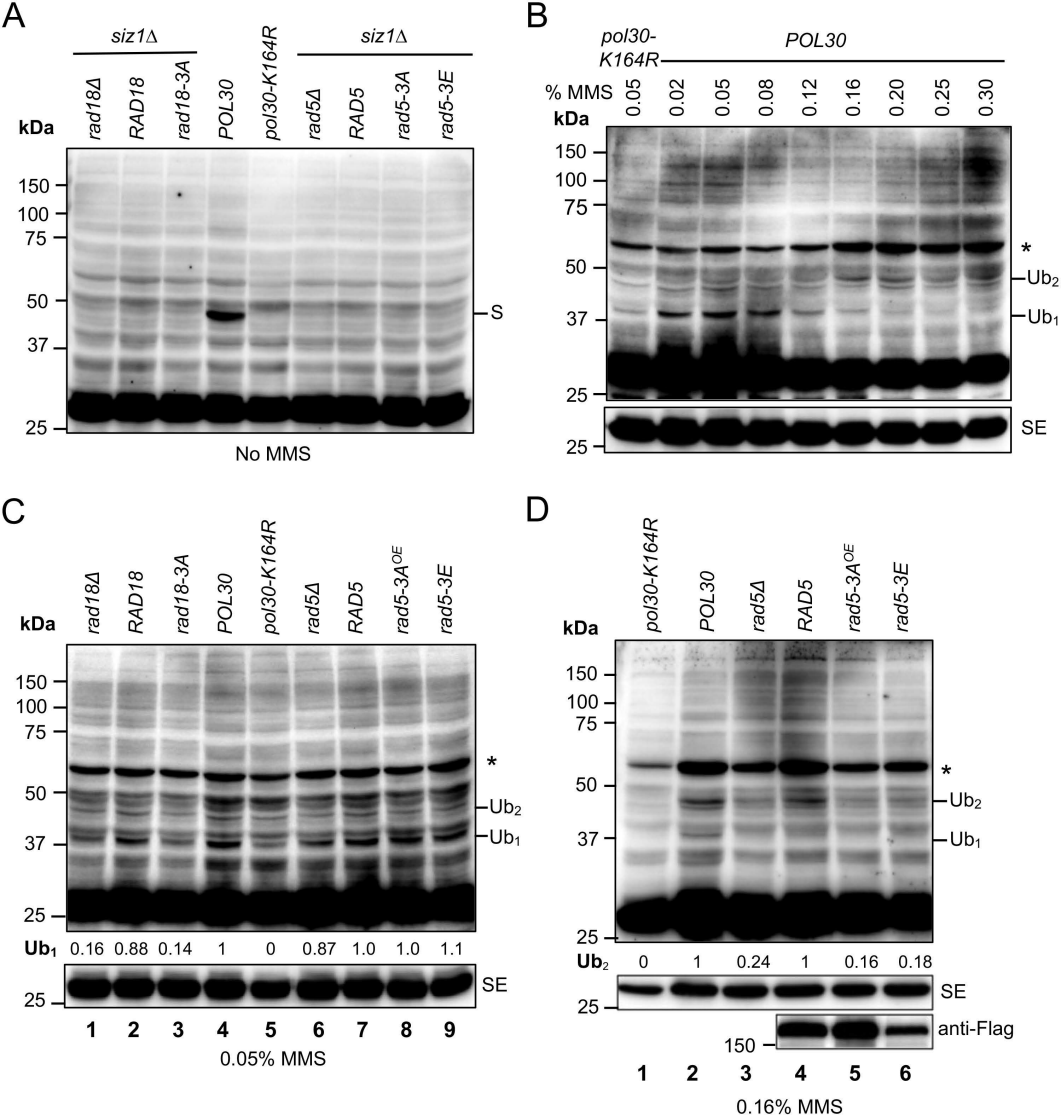
**Detection of PCNA post-translation modifications (PTMs) by western blotting**. (**A**) PCNA PTMs under no DNA damage treatment conditions. (**B**) Detection of PCNA mono- and di-ubiquitination after treatments with different MMS doses for 180 min. (**C**) Cells treated with 0.05% MMS for 180 min to assess PCNA mono-ubiquitination. (**D**) Cells treated with 0.16% MMS for 180 min to assess PCNA di-ubiquitination. All strains used are in the *siz1*∆ background except lanes 4 and 5 in (A). Abbreviations used: S, Pol30-K164^SUMO^; Ub_1_, Pol30-K164^Ub^; Ub_2_, Pol30-K164^di-Ub^; SE, short exposure; ^OE^, cells were transformed with a YEp-based plasmid. Asterisks indicate a major uncharacterized band found in all MMS treated samples. At least three independent experiments as shown in (C) and (D) were performed with multiple exposures, and a typical set of results is shown. Numbers under the main blots of (C) and (D) indicate relative intensity of mono- and di-ubiquitinated PCNA bands, respectively, normalized against corresponding anti-PCNA bands from the short exposure blots. The band intensity from the *POL30* lane was set as 1 and that from the *pol30-K164R* lane was set as 0. Unless specified, all yeast strains were in the HKY578-10D *siz1∆* background.

Since Rad18 and Rad5 are E3 Ub ligases for PCNA mono- and poly-ubiquitination, respectively 7, it raises a possibility that their interactions with PCNA play a critical role in the substrate (PCNA) ubiquitination. To test this hypothesis, a western blotting protocol tailored to detect PCNA PTMs 49 was performed using whole-cell extracts (WCEs). Although this protocol can directly visualize PCNA PTMs, the anti-PCNA antibody detected multiple non-specific bands due to the low abundance of PTMs at Pol30-K164 49. Hence, the WCE from a *pol30-K164R* mutant was used as a reference to distinguish PTMs at Pol30-K164 from other unrelated bands. Figure 6A shows that in the absence of MMS treatment, a major PCNA PTM band appeared in WT cells but was absent from *pol30-K164R* and *siz1*∆ cells, indicating that it is Pol30-K164^SUMO^.

Under our experimental conditions, Pol30-K164^SUMO^ comigrates with Pol30-K164^Ub2^; hence, a *siz1∆* mutation has been previously introduced to assess Pol30-K164 di-ubiquitination after MMS treatment 12, 50. Experimental conditions for the MMS induction of PCNA ubiquitination were examined. As shown in Figures 6B and S5A, MMS at lower doses (0.02-0.08%) mainly induced Pol30-K164 mono-ubiquitination (38 kDa), while higher MMS doses (0.25-0.3% for 90 min treatment or 0.16-0.2% for 180 min treatment) favor Pol30-K164 di-ubiquitination (47 kDa). Based on the above observations, cells treated with 0.05% MMS for 180 min were used to evaluate Pol30-K164 mono-ubiquitination, and those with 0.16% MMS for 180 min were used to evaluate Pol30-K164 di-ubiquitination. Figure 6C shows that *RAD18* transformed *rad18*∆ cells (lane 2) restored Pol30-K164^Ub1^ band comparable to *POL30* cells (lane 4), while *rad18-3A* transformed *rad18*∆ cells (lane 3) behaved like *rad18*∆ (lane 1) and *pol30-K164R* (lane 5) band patterns. In addition, the *rad5*∆ mutant and its transformants (lanes 6-9) did not display altered Pol30-K164^Ub1^ band intensity. Upon 0.16% MMS treatment (Figure 6D), *rad5*∆ (lane 3) cells displayed reduced Pol30-K164^Ub2^ band intensity in comparison to *POL30* (lane 2) and *RAD5* transformed *rad5*∆ (lane 4) cells. In contrast, YEp-*rad5-3A* (lane 5) and YCp-*rad5-3E* (lane 6) transformed *rad5*∆ cells displayed reduced Pol30-K164^Ub2^ band intensity comparable to that of *rad5*∆ cells (lane 3). Statistical analyses of data as presented in Figure 6C, D are shown in Figure S5B, C, respectively. Based on the above observations, we conclude that efficient interaction with PCNA is a prerequisite for PCNA-K164 mono-ubiquitination and poly-ubiquitination by Rad18 and Rad5, respectively.

### The Rad5 and Rad18 LxLF motif is required for the DNA damage response

**Figure 7:**
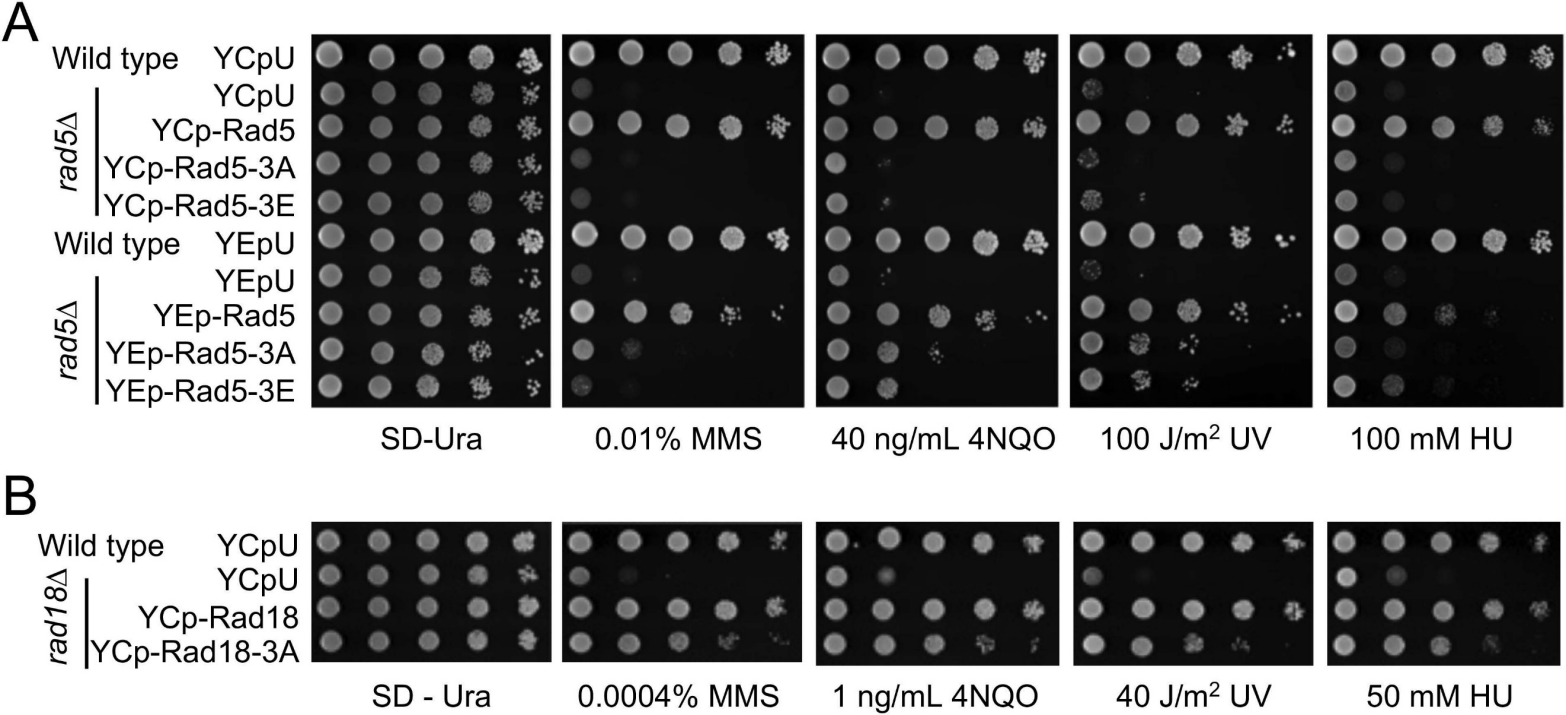
** Relative sensitivity of *rad5 and rad18* mutants to DNA-damaging agents by a serial dilution assay**. (**A**) Assessment of *RAD5* and its mutant derivatives to DNA damage response. Full-length *RAD5* along with its native promoter and terminator sequences were cloned into a YCp single-copy or YEp multi-copy plasmid and transformed into *rad5*∆ cells. Indicated amino acid substitution mutations were created to assess their effects on DNA damage response. (**B**) Assessment of *RAD18* and its *rad18-3A* mutant to DNA damage response. Full-length *RAD18* or *rad18-3A* along with their native promoter and terminator sequences were cloned into a YCp plasmid and transformed into *rad18*∆ cells. Experimental conditions were as described in Figure 1E. At least two sets of independent cultures and multiple doses of treatments were employed and only selected representative images are shown. Note that since *rad5*∆ and *rad18*∆ mutants display different levels of sensitivity, plates images with different UV doses and chemical concentrations are shown.

To evaluate whether the LxLF motif in Rad5 and Rad18 is required for their DNA-damage response, single-copy plasmids carrying *RAD5*, *RAD18* and their mutant alleles were transformed into the corresponding *rad5* and *rad18* null mutants, and the transformants were treated with MMS, 4NQO and UV that induce different types of DNA lesions with a common consequence of blocking replication, although their repair mechanisms are rather different 51. As expected, vector-transformed *rad5∆* (Figure 7A) and *rad18∆* (Figure 7B) cells are extremely sensitive to all three types of DNA-damaging agents, and their respective wild-type gene transformants can fully rescue the sensitivity. Under the above experimental conditions, the *rad5-3A* and *rad5-3E* transformants failed to restore the DDT activity (Figure 7A). To address a concern that *rad5-3A* and *rad5-3E* reduced cellular Rad5 protein levels (Figure 5C), we also transformed *rad5*∆ cells with YEp-based plasmids, in which YEp-rad5-3A and YEp-rad5-3E transformants produced Rad5 proteins no less than the YCp-RAD5 transformed cells under both untreated and MMS-treated conditions (Figure S3A-C). Figure 7A shows that overexpression of *rad5-3A* or *rad5-3E* still did not fully restore the *RAD5* activity. On the other hand, the *rad18-3A* transformant did not display reduced Rad18 protein level (Figure 5D) but was more sensitive to DNA-damaging agents than *RAD18* transformed cells (Figure 7B).

To ask whether overexpression of *rad18-3A* can compensate for its partial loss-of-function defect, we transformed *rad18*∆ cells with a pGAD424-based multicopy plasmid in which the cloned gene is driven by a strong *ADH1* promoter. While overexpression of *GAL4_AD_-RAD18* fully complemented the *rad18*∆ defect, *GAL4_AD_-rad18-3A* behaved like the null mutant (Figure S6A). Similarly, overexpression of *GAL4_AD_-rad5-3A* had little if any effects on functional complementation of *rad5*∆ (Figure S6B). In both cases, a possibility that the 3A mutations compromise the fusion protein stability has been effectively ruled out, as these mutations did not affect physical interaction with other Rad5/Rad18 interaction proteins in a Y2H assay (Figure 5A, B).

It was recently reported that Rad5 is required for DNA synthesis across undamaged templates 52. To ask whether this function requires PCNA interaction, we assessed relative sensitivities of wild-type, *rad5*∆, *rad5-3A* and *rad5-3E* cells to hydroxyurea (HU) that inhibits ribonucleotide reductase activity, depletes the dNTP pool and stalls replication forks in the absence of DNA damage 53. Indeed, *rad5*∆ cells displayed increased sensitivity to HU as anticipated, while *rad5-3A* and *rad5-3E* mutants behave like the null mutant, regardless of Rad5 protein levels (Figure 7A), indicating that binding to PCNA is absolutely required by Rad5 for this function. Interestingly, *rad18∆* cells also displayed increased sensitivity to HU, while *rad18-3A* cells are partially sensitive to HU (Figure 7B). Hence, interaction with PCNA is not absolutely required by Rad18 to participate in DNA synthesis across undamaged templates.

## Discussion

In this study, we identified and functionally characterized a novel PCNA-binding motif from budding yeast Rad18 and Rad5, two Ub ligases required for PCNA mono- and poly-ubiquitination, respectively. Firstly, since E3 ligases are responsible for the substrate recognition, they must contain PCNA-binding domain(s). Through sequential truncations, we separately mapped PCNA-binding regions to within 40 residues in both Rad5 and Rad18, from which a putative LxLF motif was identified. Secondly, Y2H, *in vitro* affinity pulldown and *in vivo* co-IP assays collectively confirm that this LxLF-containing sequence is required and sufficient for the Rad5/Rad18 interaction with PCNA, which is consistent with and advances previous reports 7, 37, 38. Thirdly, 3A substitutions in the Rad5 and Rad18 LxLF motifs specifically compromise the interaction with PCNA but not other binding partners; meanwhile, Rad18-3A abolishes both MMS-induced PCNA mono-ubiquitination and di-ubiquitination, while Rad5-3A affects MMS-induced PCNA di-ubiquitination but not mono-ubiquitination, confirming that binding to their substrate PCNA is a prerequisite for the substrate PTM by these two RING E3s. Fourthly, it was found that substitution of the LxLF motif in Rad5 leads to a severely compromised DDT phenotype, while substitution of the LxLF motif in Rad18 partially affects its DDT function. Furthermore, these loss-of-function phenotypes cannot be offset by increasing cellular mutant protein levels, indicating that the 3A substitutions are not simply partial loss-of-function mutations. Finally, it is noted that although the Rad18-3A substitution does not affect the protein stability *in vivo* and *in vitro*, it reduced PCNA mono-ubiquitination to a level indistinguishable from that of *rad18*∆; yet, the *rad18-3A* cells only displayed partial sensitivity to DNA-damaging agents. One possible explanation is that Rad18 may contain more than one region to contact PCNA, or may be recruited to the stalled replication fork by interacting with other proteins like Rad5 43 and SUMO 44, or ssDNA 54, 55, while our WB analysis was not sensitive enough to detect residual amount of ubiquitinated PCNA in *rad18-3A* cells. Alternatively, *rad18-3A* mutant cells may still be able to support a partial DDT activity independently of PCNA mono-ubiquitination. For example, it was recently reported that a Rad5-mediated mutagenic repair is independent of Rev1-PCNA interaction 56, which may require Rad18 but does not require PCNA binding and mono-ubiquitination.

We 47 and others 48 previously reported that the Rad5 HIRAN domain may be required for its interaction with PCNA. In addition to the internal deletion 47, a HIRAN-3RE mutation 48 also compromises Rad5-PCNA interaction. Since a Rad5-K194E substitution within HIRAN inactivates its DNA-binding and enzymatic activities 45, 46 but does not affect PCNA binding, its enzymatic activity does not appear to be required for the PCNA interaction. Interestingly, the Rad5 HIRAN domain is adjacent to the LxLF motif, raising a possibility that they jointly contribute to PCNA binding. However, a reported *Kl*Rad5 crystal structure 48 shows that a mediator helix harboring the MxLF motif is buried between HIRAN and Snf2 domains, making it unlikely to contact PCNA. Furthermore, the AlphaFold modeling predicts the HIRAN domain makes contact to the back face of PCNA (Figure S8A). However, we have not been able to demonstrate that the HIRAN domain alone binds PCNA despite repeated attempts. In contrast, the Rad5 LxLF-containing 40-aa region without HIRAN is sufficient to bind PCNA. One possibility remains that when associated with DNA/chromatin via HIRAN/Snf2 domains, Rad5 may undergo conformational change to expose this mediator helix for the PCNA interaction. Alternatively, this LxLF-containing mediator helix may not contact PCNA as predicted by AlphaFold; consequently, the Rad5-3A substitution may affect PCNA binding and other related DDT functions due to compromised structural integrity.

The available human Rad18 RING structure 57 allowed us to predict that the embedded Rad18 LxLF motif and its flanking residues are exposed, and the AlphaFold-predicted PCNA-Rad18 complex indicates that the LxLF-containing region, together with an extended loop from residues 270-280 of Rad18, contact PCNA on its back face (Figure S8B). In this case, we suspect that the RING domain is not required for binding PCNA but plays a critical role in maintaining structural integrity of full-length Rad18.

Although the AlphaFold modeling does not reveal structural conservation between LxLF-containing regions from Rad5 and Rad18, its predicted Rad5-PCNA and Rad18-PCNA complex structures, together with this study jointly support a notion that both Rad5 and Rad18 share a binding surface on PCNA distinct from that of PIP-box proteins. Interestingly, many well-studied yeast proteins involved in DNA metabolism including replicative and TLS polymerases contain PIP boxes and bind to the front face of PCNA 17, 58, whereas K168 and K196 are adjacent to PCNA-K164 located on the back face (Figure 1A). One can imagine a scenario that when a replicative polymerase is stalled due to replication block or depleted dNTP pool, Rad18 can sense the signal and mono-ubiquitinate PCNA at its back face, which recruits TLS polymerases like Polη/Rad30 that contains a PIP box 40, 59 and a Ub-binding domain UBZ 60. Another TLS polymerase Rev1 also contains a Ub-binding domain UBM but does not contain a PIP box, and its N-terminal BRCT domain has been reported to be required for PCNA binding 61, 62, although its binding surface on PCNA remains unclear. Meanwhile, Rad5 cross talks with Rev1 through its N-terminus 38. Hence, the spatial arrangement between PCNA E3 ligases and DNA polymerases may facilitate polymerase switch 63 in response to DNA damage.

The LxLF motif appears to be conserved only in certain species of lower eukaryotes (Supplementary Figure S7). In Rad5, the first Leu residue may be replaced by a conserved residue in some species. For example, in *Kl*Rad5, whose Rad5 crystal structure is resolved 48, the first Leu residue is replaced by Met (Figure S7A). In Rad18, the Phe residue in the LxLF motif appears to be variable among these species, although their flanking RING domains are highly conserved (Figure S7B). Mammalian Rad5 and Rad18 homologs do not appear to contain a conserved LxLF motif. Instead, two human Rad5 homologs HLTF and SHPRH contain an APIM motif responsible for the PCNA interaction 64. The PCNA-binding region in hRad18 has been previously mapped to aa 16-366 54, although the exact PCNA-binding sequence has not been determined. In contrast, it was recently reported 65 that hRad18 contains a cryptic PIP motif at aa 402-410 responsible for binding PCNA; however, budding yeast Rad18 does not contain such a cryptic PIP consensus sequence. Although the LxLF motif appears to be required by both Rad5 and Rad18 to interact with PCNA, this motif alone is unlikely sufficient to bind PCNA. We anticipate that additional flanking sequence and structural analyses are needed to define this novel LxLF-containing PCNA-binding domain and notice that AlphaFold in combination with NMR and cross-linking mass spectrometry has been successfully employed to predict hRad18 structure in complex with its binding partners 66. Once the Rad5/Rad18-PCNA complex structures are revealed and/or additional consensus sequences are identified, one can search for the yeast and possibly other eukaryotic genomes for proteins containing this newly defined PCNA-binding domain.

## Supplementary Material

Supplementary figures and tables.

## Data Availability

All the data supporting the findings of this study are available within the article and supplementary information files or can be obtained from the corresponding author upon reasonable request.
