# Supplementary material for: Budding Yeast Ubiquitin Ligases Rad5 and Rad18 Bind a Novel PCNA Surface, which Is Required for their Functions in DNA-Damage Tolerance
Source: Int J Biol Sci. 2026 Feb 26;22(6):3032–47. doi: 10.7150/ijbs.124441 (PMC13050448; doi:10.7150/ijbs.124441)
Supplement: Supplementary file 1 — Supplementary figures and tables. [file ijbsv22p3032s1.pdf]

Supplementary Material

Table S1. *Saccharomyces cerevisiae* strains used in this study

Strain	Genotype	Source
**HK578-10D**	*MATα*, *leu2-3,112, trp1-1, can1-100, ura3-1, ade2-1, his3-11,15*	H. Klein
**WXY971**	HK578-10D with *rad18Δ::TRP1*	Lab stock
**WXY2561**	HK578-10D with *rad5Δ::TRP1*	This study
**HK578-10A**	*MATa*, *leu2-3,112 trp1-1, can1-100, ura3-1, ade2-1, his3-11,15*	H. Klein
**WXY2562**	HK578-10A with *rad18Δ::TRP1*	This study
**WXY2563**	HK578-10A with *rad5Δ::TRP1*	This study
**WXY939**	HK578-10D with *pol30Δ::HIS3*/pBL211	Lab stock
**WXY2972**	WXY939 with *rad5Δ::TRP1*	Lab stock
**WXY978**	WXY939 with *rad18Δ::TRP1*	Lab stock
**WXY2558**	WXY939 with *siz1Δ::LEU2*	This study
**WXY2559**	WXY2972 with *siz1Δ::LEU2*	This study
**WXY2560**	WXY978 with *siz1Δ::LEU2*	This study
**PJ69-4a**	*MATa*, *trp1-901 leu2-3,112 ura3-52, his3-200, gal4Δ, gal80Δ, GAL2-ADE2 LYS2::GAL1-HIS3, MET2::GAL7-lacZ*	P. James

Table S2. Oligonucleotides used in this study.

Name	Sequence (5'-3')
**pGBT-Pol30-1-258-*Bam*HI-F**	CGCCGCGGATCCATGTTAGAAGCAAAATTTG
**pGBT-Pol30-1-258-*Pst*I-R**	GCAGTTATTCTTCGTCATTAAATTTAGGAGCC
**pGBT/AD-Rad5-1-1167-*Bam*HI-F**	CGCCGCGGATCCATGAGTCATATTGAACAGG
**pGBT/AD-Rad5-1-1167-*Pst*I-R**	GCATGCACTGCAGCTATTCAAACAGCATCTGGATTTC
**pGBT-Rad5-HIRAN-*Bam*HI-F**	CGCCGCGAATCCATGATAGGTGCTTTGCAAGTCACTGG
**pGBT-Rad5-HIRAN-*Pst*I-R**	TGCACTGCAGTTAGCAATCCAATTGTAGAATAAAGC
**pGBT-Rad5-1-100-*Bam*HI-F**	CGCCGCGGATCCATGAGTCATATTGAACAGG
**pGBT-Rad5-1-100-*Pst*I-R**	TGCACTGCAGTTATTTTTCATTAAGTTCCATCAC
**pGBT-Rad5-100-200-*Bam*HI-F**	CGCCGCGGATCCATGAAATTCGGCAGTCAGGAGG
**pGBT-Rad5-100-200-*Pst*I-R**	GCATGCACTGCAGTTACTTCATCTGAGAGCCGTACTTC
**pGBT-Rad5-200-300-*Bam*HI-F**	CGCCGCGGATCCATGAAGCTAAAGAGATCAAGTGAAGAGA
**pGBT-Rad5-200-300-*Pst*I-R**	TGCACTGCAGGGATTCTCCATCATTACGTTCCTC
**pGBT-Rad5-300-400-*Bam*HI-F**	CGCCGCGGATCCATGTCCTTGATGAAAAGAAGACG
**pGBT-Rad5-300-400-*Pst*I-R**	GCACTGCAGTTATTCTTCTTCGTGCTGAGTATCTC
**pGBT-Rad5-400-431-*Bam*HI-F**	CGCCGCGGATCCATGGAAACAATGAACTTGAATCAATTG
**pGBT-Rad5-400-431-*Pst*I-R**	TGCACTGCAGATCGCGAGAAGGTTCTGTTTCAGGC
**pGBT-Rad5-300-360-*Bam*HI-F**	CGCCGCGGATCCATGTCCTTGATGAAAAGAAGACG
**pGBT-Rad5-300-360-*Pst*I-R**	TGCACTGCAGTTATAATGCCTTCTGCTCGTCC
**pGBT-Rad5-340-360-*Bam*HI-F**	CGCCGCGGATCCATGGCTCTACTAAAGTTATTTG
**pGBT-Rad5-340-360-*Pst*I-R**	TGCACTGCAGTTATAATGCCTTCTGCTCGTCC
**pGBT-Rad5-320-380-*Bam*HI-F**	CGCCGCGGATCCATGAATTTTGGAAGAACATTGACTG
**pGBT-Rad5-320-380-*Pst*I-R**	TGCACTGCAGTTACTCGTCGTTATCTAAATCAATGATTTC
**pGBT-Rad5-100-431-*Bam*HI-F**	CGCCGCGGATCCATGAAATTCGGCAGTCAGGAGG
**pGBT-Rad5-100-431-*Pst*I-R**	TGCACTGCAGATCGCGAGAAGGTTCTGTTTCAGGC
**pGBT-Rad5-300-340-*Bam*HI-F**	CGCCGCGGATCCATGTCCTTGATGAAAAGAAGACG
**pGBT-Rad18-300-340-*Pst*I-R**	TGCACTGCAGTTACAGTCTTTTCGAGCGGC
**pGBT-Rad5-320-360-*Bam*HI-F**	CGCCGCGGATCCATGAATTTTGGAAGAACATTGACTG
**pGBT-Rad5-320-360-*Pst*I-R**	TGCACTGCAGTTATAATGCCTTCTGCTCGTCC
**pGBT-Rad5-340-380-*Bam*HI-F**	CGCCGCGGATCCATGGCTCTACTAAAGTTATTTG
**pGBT-Rad5-340-380-*Pst*I-R**	CTGCAGTTACTCGTCGTTATCTAAATCAATGATTTC
**pGBT-Rad5-360-400-*Bam*HI-F**	CCGCCGCGGATCCATGTTAGAAAAGCATAAAATAGAGC
**pGBT-Rad5-360-400-*Pst*I-R**	GCACTGCAGTTATTCTTCTTCGTGCTGAGTATCTC
**pGBT-Rad5-171-348-*Bam*HI-F**	CGCCGCGGATCCATGACTTGGAAAAGATTTATAGGTGC

**pGBT-Rad5-171-348-*Pst*I-R**	GCATGCACTGCAGTTACCTCAATTTATCAAATAAC
**pGBT-Rad5-1-321-*Bam*HI-F**	CGCCGCGGATCCATGAGTCATATTGAACAGG
**pGBT-Rad5-1-321-*Pst*I-R**	GCAGTTATCCAAAATTTCCATTGTCTTTCTCTC
**pGBT/AD-Rad18-1-348-*Bam*HI-F**	CGCCGCGGATCCATGGACCACCAAATAACCACTGCAAGCG
**pGBT/AD-Rad18-1-348-*Pst*I-R**	CATGCACTGCAGCAGTGGTAATATATCAGTCATGGATCTTTTGGC
**pGBT-Rad18-1-160-*Bam*HI-F**	CGCCGCGGATCCATGGACCACCAAATAACCACTGCAAGCG
**pGBT-Rad18-1-160-*Pst*I-R**	GCATGCACTGCAGTTACAGTGGTAATATATCAGTCATGG
**pGBT-Rad18-150-258-*Bam*HI-F**	CGCCGCGGATCCGCCAAAAGATCCATGACTGATATATTACCACT
**pGBT-Rad18-150-258-*Pst*I-R**	ATGCACTGCAGCACATGTGAGGTCTCACCACATGAGCTTTTGTCG
**pGBT-Rad18-240-371-*Bam*HI-F**	CGCCGCGGATCCTTCAAGGTACGAACTCCAGAAGTCGACAAAAG
**pGBT-Rad18-240-371-*Pst*I-R**	GCATGCACTGCAGCCTATATGAAGAAGATTTCCC
**pGBT-Rad18-300-487-*Bam*HI-F**	CGCCGCGGATCCACTAGGCAAAACATGATTAAAAGATACAATCA CTAC
**pGBT-Rad18-300-487-*Pst*I-R**	GCATGCACTGCAGTTAATTGTTACCGGGTGGGTC
**pGBT-Rad18-1-40-*Bam*HI-F**	CGCCGCGGATCCATGGACCACCAAATAACCACTGCAAGCG
**pGBT-Rad18-1-40-*Pst*I-R**	GCATGCACTGCAGTTATAAGACGGGGACTTTTAG
**pGBT-Rad18-20-60-*Bam*HI-F**	CGCCGCGGATCCATGTACCAATTGGATACACTTTTGAG
**pGBT-Rad18-20-60-*Pst*I-R**	GCATGCACTGCAGTTATGGTTGGTTATTCAAATGTG
**pGBT-Rad18-40-80-*Bam*HI-F**	CGCCGCGGATCCATGTTAACACCTTGTGGCCATAC
**pGBT-Rad18-40-80-*Pst*I-R**	GCATGCACTGCAGTTAGACCAGGAACTCACTTCTCAGC
**pGBT-Rad18-60-100-*Bam*HI-F**	CGCCGCGGATCCATGCCAAATTGTCCTCTCTGCCTTTTCGAG
**pGBT-Rad18-60-100-*Pst*I-R**	GCATGCACTGCAGTTATATCCTTAGTGCATCTAG
**pGBT-Rad18-80-120-*Bam*HI-F**	CGCCGCGGATCCATGGTCAGTGAAATAATTCAAAG
**pGBT-Rad18-80-120-*Pst*I-R**	GCATGCACTGCAGTTACCATGAAGAATTTTCAGGACCTGG
**pGBT-Rad18-100-140-*Bam*HI-F**	CGCCGCGGATCCATGATACCGAAGCCTACCCCTGTCCC
**pGBT-Rad18-100-140-*Pst*I-R**	GCATGCACTGCAGTTATTGCAAGTCATCATCAGCGGC
**pGBT-Rad18-120-160-*Bam*HI-F**	CGCCGCGGATCCATGTGGATAGAACTCATATCAGAGTCTG
**pGBT-Rad18-120-160-*Pst*I-R**	GCATGCACTGCAGTTACAGTGGTAATATATCAGTCATGG
**Pol30-*Bam*HI-F**	CGCGGATCCATGTTAGAAGCAAAATTTGAAGAAGCATCCC
**Pol30-*Sac*I-R**	CGAGCTCTTATTCTTCGTCATTAAATTTAGGAGCC
**Rad5-N431-*Bam*HI-F**	CGCCGCGGATCCATGAGTCATATTGAACAGGAAGAAAGGAAGA
**Rad5-N431-*Eco*RI-R**	CCGGAATTCTTAATCGCGAGAAGGTTCTGTTTCAGGC
**Rad18-N160-*Bam*HI-F**	CGCCGCGGATCCATGGACCACCAAATAACCACTGCAAGCG
**Rad18-N160-*Xho*I-R**	CCGCTCGAGTTACAGTGGTAATATATCAGTCATGG
**Rad30-*Bam*HI-F**	CGCCGCGGATCCATGGTTGTAGATATGTTTGGCAATCAGG
**Rad30-*Eco*RI-R**	CCGGAATTCTCATTTTTTTCTTGTAAAAAATGATAAGATG

**Rad18-*Sac*I-F**	CGAGCTCGGAAGCGCGCAACTGAAAAG
**Rad18-*Bam*HI-R**	CGCGGATCCATTGTTACCGGGTGGGTCTTTAC
**2×Flag-*Bam*HI-F**	CGCGGATCCATGGATTACAAGGATGACGACGATAAGG
**2×Flag-*Sph*I-R**	ACATGCATGCTTACTTGTCATCATCGTCCTTATAG
**Rad18-*Sph*I-F**	ACATGCATGCGGTGTGTATATGTAATCAGGCCTG
**Rad18-*Hind*III-R**	CCCAAGCTTCACTTCTTTGCCTAATCCAGTGG
**Rad5-*Bam*HI-F**	CGCGGATCCCCATCAAAAGAAGACAAGACCTGGAG
**Rad5-S1-R**	CCTGTTCAATATGACTCATCTTGTCATCATCGTCCTTATAGTCCTT ATCGTCGTCATCCTTGTAATCCATATTGTGATACTGTAAGACTTT AGGTGTG
**Rad5-S2-F**	CACACCTAAAGTCTTACAGTATCACAATATGGATTACAAGGATG ACGACGATAAGGACTATAAGGACGATGATGACAAGATGAGTCAT ATTGAACAGG
**Rad5-S2-R**	GCCATCTTCAACGTTTACATGGTCCTGCTGC
**Rad5-S3-F**	GCAAGGTCTTACTTGGATGCTAAGGAGGGAGC
**Rad5-S3-R**	GGTTCCGTTGTGCAGATGGAGCACTCTAAGGACTG
**Rad5-S4-F**	GGTTACGGAACAAACGGTGGAGCTTGACTC
**Rad5-*Kpn*I-R**	CGGGGTACCAAAAAATCTACTAGTTTATTTCCACCTACAC
**Pol30-K168-F**	GATCACCAAAGAAACAATAGCGTTTGTAGCTGACGGTGATATC GGATC
**Pol30- K168-R**	CCGTCAGCTACAAACGCTATTGTTTCTTTGGTGATCATGATATTA ATAG
**Pol30-K196A-F**	CCTGAAACAAGCATCGCACTTGAAATGGATCAACCTGTCGACTT GACGTTCGG
**Pol30-K196A-R**	CATTTCAAGTGCGATGCTTGTTTCAGGATGTTCCATATCCACGAA TGGTTTTATTATGAC
**Pol30-K164R-F**	GATTCTATTAATATCATGATCACCAGAGAAACAATAAAGTTTGT AGCTGACGGTGATATCG
**Pol30-K164R-R**	CTTTATTGTTTCTCTGGTGATCATGATATTAATAGAATCACTCAA TTGGGACAAG
**Pol30-K126,128AA-R**	CTGTAATTCTTCCGCCTTCGCGAAATCAGCATCGATATCCATC
**Pol30-251-255-AAA-F**	CAGTTTTTCTTGGCTGCGGCGGCGGCTGACGAAGAATAA
**Pol30-251-255-AAA-R**	TTATTCTTCGTCAGCCGCCGCCGCAGCCAAGAAAAACTGTAGGA ACCCACTC
**Rad5-LLF341,343, 344AAA-F (Rad5-3A-F)**	GAAAGCCGCTCGAAAAGACTGGCTCTAGCGAAGGCGGCGGATA AATTGAGGCTAAAGCC

**Rad5-LLF341,343,344AAA-R (Rad5-3A-R)**	CCAAAATAGGCTTTAGCCTCAATTTATCCGCCGCCTTCGCTAGAGCCAGTCTTTTCGAGC
**Rad5-L341A-F**	GAAAGCCGCTCGAAAAGACTGGCTCTAGCGAAGTTATTTGATAAATTGAGGCTAAAGCC
**Rad5-L341A-R**	CCAAAATAGGCTTTAGCCTCAATTTATCAAATAACTTCGCTAGAGCCAGTCTTTTCGAGC
**Rad5-L343A-F**	GAAAGCCGCTCGAAAAGACTGGCTCTACTAAAGGCGTTTGATAAATTGAGGCTAAAGCC
**Rad5-L343A-R**	CCAAAATAGGCTTTAGCCTCAATTTATCAAACGCCTTTAGTAGAGCCAGTCTTTTCGAGC
**Rad5-F344A-F**	GAAAGCCGCTCGAAAAGACTGGCTCTACTAAAGTTAGCGGATAAATTGAGGCTAAAGCC
**Rad5-F344A-R**	CCAAAATAGGCTTTAGCCTCAATTTATCCGCTAACTTTAGTAGAGCCAGTCTTTTCGAGC
**Rad5-R187E-F**	GCTTTGCAAGTCACTGGTATGGCTACCGAACCCACCGTCAGG
**Rad5-R187E-R**	GCCGTACTTCAAGGGCCTGACGGTGGGTTCGGTAGCCATACC
**Rad5-RR229,241EE-F (Rad5-2E-F)**	GAAAGCGTCCATGGCTAGTTTGGTAGAAATTTTTGATATCCAATATGATAGAGAAATTGGCGAAGTTTCGGAAGAC
**Rad5-RR229,241EE-F (Rad5-2E-R)**	GTATTTGAGCAATGTCTTCCGAAACTTCGCCAATTTCTCTATCATATTGGATATCAAAAATTTCTACCAAACTAGC
**Rad18-LLF64,66,67AAA-F (Rad18-3A-F)**	CATTTGAATAACCAACCAAATTGTCCTGCGTGCGCGGCGGAGTTCAGAGAGTCCTTGCTG
**Rad18-LLF64,66,67AAA-R (Rad18-3A-R)**	AACTCACTTCTCAGCAAGGACTCTCTGAACTCCGCCGCGCACGCAGGACAATTTGGTTGG
**Rad18-L64A-F**	CATTTGAATAACCAACCAAATTGTCCTGCGTGCCTTTTCGAGTTCAGAGAGTCCTTGCTG
**Rad18-L64A-R**	AACTCACTTCTCAGCAAGGACTCTCTGAACTCGAAAAGGCACGCAGGACAATTTGGTTGG
**Rad18-L66A-F**	CATTTGAATAACCAACCAAATTGTCCTCTCTGCGCGTTCGAGTTCAGAGAGTCCTTGCTG
**Rad18-L66A-R**	AACTCACTTCTCAGCAAGGACTCTCTGAACTCGAACGCGCAGAGAGGACAATTTGGTTGG
**Rad18-F67A-F**	CATTTGAATAACCAACCAAATTGTCCTCTCTGCCTTGCGGAGTTCAGAGAGTCCTTGCTG
**Rad18-F67A-R**	AACTCACTTCTCAGCAAGGACTCTCTGAACTCCGCAAGGCAGAGAGGACAATTTGGTTGG

**Rad5-K194E-F**	GGCTACCAGACCCACCGTCAGGCCCTTGGAGTACGGCTCTCAGATG
**Rad5-K194E-R**	CACTTGATCTCTTTAGCTTCATCTGAGAGCCGTACTCCAAGGGCCTGACG
**Rad18-C28C-F**	GCCTGTACCAATTGGATACACTTTTGAGAAGTCACATTTGTAAAGATTTTCTAAAAG
**Rad18-C28C-R**	GACGGGGACTTTTAGAAAATCTTTACAAATGTGACTTCTCAAAAGTGTATCC
**Rad18-C65S-F**	GAATAACCAACCAAATTGTCCTCTCAGCCTTTTCGAGTTCAGAGAG
**Rad18-C65S-R**	CAGCAAGGACTCTCTGAACTCGAAAAGGCTGAGAGGACAATTTGGTTGG

Supplementary Figure Legends

Figure S1. Relative sensitivity of *pol30* mutants to DNA-damaging agents by a serial dilution assay. The *pol30* mutants were created by plasmid shuffling. Cells were cultured overnight, density adjusted, serially diluted by tenfold and spotted onto YPD plates containing indicated concentrations of chemicals. For the UV irradiation, the plates were exposed to UV in a UV crosslinker at the given dose. The plates were incubated at 30 ^{o}C for two days before photography. At least two sets of independent cultures and multiple doses of DNA damage were employed and only selected representative images are shown.

Figure S2. Assessment of amino acid substitutions in Rad5 and Rad18 by a Y2H assay. (**A**) Effects of amino acid substitutions in the Rad5 LxLF motif. (**B**) Effects of amino acid substitutions in the Rad18 LxLF motif. (**C**) Effects of amino acid substitutions in the Rad18 RING domain. The Quick Exchange method was used to create site-specific mutations that result in single amino acid substitutions. Experimental conditions were as described in Figure 1B.

Figure S3. **Assessment of cellular Rad5 and Rad18 protein levels by western blotting**. (**A**) Relative Rad5 protein levels from *rad5*Δ cells transformed with either YCp-Flag-Rad5 or YEp-Flag-Rad5 without (upper panel) or with 0.05% MMS (lower panel) treatment. Experimental conditions were as described in Fig. 5C. (**B**, **C**) Quantitative analysis of western blot data as shown in (A). (**B**) No treatment. **(C)** 0.05% MMS treatment. (**D**, **E**) Quantitative analysis of western blot data as shown in Fig. 5C and D. (**B-E**) At least three independent western blots from each set of experiment were used for the quantitative analysis. The results are presented as average relative protein levels $\pm$ standard deviations shown as error bars (n=3).

Figure S4. Additional western blot results from the *in vivo* co-IP assays. (**A**, **B**) Physical interaction between Rad5 and Pol30. (**C**, **D**) Physical interaction between Rad18 and Pol30. Experimental conditions were as described in Figure 5E and G.

Figure S5. Characterization of PCNA post-translation modifications. (**A**) Western blotting to assess effects of 90-min MMS treatments with different doses on PCNA ubiquitination. All strains are in the *siz1*Δ background. Abbreviations used: Ub_1, Pol30-K164^{Ub}; Ub_2, Pol30-K164$^{di\text{-}Ub}$; SE, short exposure. The asterisk indicates a major uncharacterized band found in all MMS treated samples Experimental conditions were as described in Figure 6B. (**B**) Quantitative analysis of mono-ubiquitinated PCNA as shown in Figure 6C. (**C**) Quantitative analysis of di-ubiquitinated PCNA as shown in Figure 6D. (**B**, **C**) At least three independent western blots from each set of experiment were used for the quantitative analysis. The results are presented as average relative protein levels ± standard deviations shown as error bars (n=3). Two-tailed *P* values from the unpaired *t*-test are shown.

Figure S6. Relative sensitivity of *rad18* (A) and *rad5* (B) mutants to DNA-damaging agents by a serial dilution assay. *RAD5* and *RAD18* were cloned in a multicopy plasmid pGAD424 with an *ADH1* promoter that produces $Gal4_{AD}$ fusion proteins. At least two sets of independent cultures and multiple doses of treatments were employed and only selected representative images are shown.

Figure S7. Amino acid sequence alignments of Rad5 (A) and Rad18 (B) in selected yeast species. Residues highlighted by black are identical sequences and those by grey are conserved sequences. Putative LxLF motifs are labelled, and conserved residues are indicated by red asterisks, while conserved Cys residues in the Rad18 RING domain are indicated by blue asterisks.

Figure S8. Predicted Rad5-PCNA and Rad18-PCNA structures. Molscript [1] was used to draw the ribbons representation. The AlphaFold3-predicted PCNA trimeric ring is shown in gray. Each subunit is colored in a different shade of gray. The ring is viewed from its side with its back face on the left. (**A**) Rad5-PCNA. Residues 1-169 of Rad5 are shown in yellow. The HIRAN domain (residues 170-300) is colored cyan. The intermediate region (residues 301-429) is shown in blue. The helicase domain (residues 430-1169) is colored in green with the embedded RING domain (residues 910-990) in darker green. (B) Rad18-PCNA. Residues 1-99 corresponding to the Rad18

RING Finger domain are shown in blue. Residues 270-280 (shown in cyan) were predicted to make intimate contact with PCNA. The rest of the Rad18 were colored yellow.

Methods and Results:

Modeling of protein complexes using AlphaFold 2 and AlphaFold 3

Both ColabFold [2], which is based on based on AlphaFold 2 [3], and AlphaFold 3 [4] were used to build models for PCNA-Rad5 and PCNA-Rad18 complexes. ColabFold was employed first to predict the two complexes. The five predicted models were consistent for PCNA-Rad5 and for PCNA-Rad18 complexes. We then used ColabFold to build Rad5 and Rad18 complexes with PCNA trimer. In both cases, PCNA trimers were predicted as closed rings, and the resulting interfaces with Rad5 and Rad18 were both consistent with predicted interfaces when monomeric PCNA was modeled. When AlphaFold 3 became available, the same procedure was repeated using AlphaFold 3. The predicted Rad5-PCNA interfaces were consistent with ColabFold predictions when PCNA monomer or trimer were modeled. The predicted Rad18-PCNA monomer interface was also consistent with ColabFold results. However, AlphaFold 3 predicted no interaction between Rad18 and closed trimeric ring of PCNA. This is not surprising since transient protein-protein interfaces remain challenging for the structural prediction.

Molscript [1] was used to generate molecular graphics. One of the five models of Rad5-PCNA trimer built by AlphaFold3 was visualized. In this AlphaFold 3-predicted model, Rad5 approaches the PCNA trimeric ring from its back face. The HIRAN domain (in cyan) is the only domain of Rad5 that makes contact with PCNA (in gray). The N-terminal side of the HIRAN domain (in yellow) is largely disordered. The LxLF motif (in red) is in the intermediate region (in blue) between the HIRAN domain and the helicase domain (in green). One of the five models of Rad18-PCNA trimer built by ColabFold was also visualized. In this predicted model, Rad18 also approaches the PCNA trimeric ring from its back face. The N-terminal RING Finger domain (in blue), which harbours the LxLF motif (in red), contacts PCNA (in gray). The rest of the predicted Rad18 structure (in yellow) is largely disordered but part of it does appear to fold around the RING Finger domain as the structural core. An extended peptide from residue 270 to 280 (in cyan) makes intimate contact with PCNA.

References:

1 Kraulis PJ. A program to produce both detailed and schematic plots of protein structures. J. Appl. Cryst. 1991; 24: 946-50.

2 Mirdita M, Schutze K, Moriwaki Y, Heo L, Ovchinnikov S, Steinegger M. ColabFold: making protein folding accessible to all. Nat Methods. 2022; 19: 679-82.

3 Jumper J, Evans R, Pritzel A, Green T, Figurnov M, Ronneberger O, Tunyasuvunakool K, Bates R, Zidek A, Potapenko A, Bridgland A, Meyer C, Kohl SAA, Ballard AJ, Cowie A, Romera-Paredes B, Nikolov S, Jain R, Adler J, Back T, Petersen S, Reiman D, Clancy E, Zielinski M, Steinegger M, Pacholska M, Berghammer T, Bodenstein S, Silver D, Vinyals O, Senior AW, Kavukcuoglu K, Kohli P, Hassabis D. Highly accurate protein structure prediction with AlphaFold. Nature. 2021; 596: 583-9.

4 Abramson J, Adler J, Dunger J, Evans R, Green T, Pritzel A, Ronneberger O, Willmore L, Ballard AJ, Bambrick J, Bodenstein SW, Evans DA, Hung CC, O'Neill M, Reiman D, Tunyasuvunakool K, Wu Z, Zemgulyte A, Arvaniti E, Beattie C, Bertolli O, Bridgland A, Cherepanov A, Congreve M, Cowen-Rivers AI, Cowie A, Figurnov M, Fuchs FB, Gladman H, Jain R, Khan YA, Low CMR, Perlin K, Potapenko A, Savy P, Singh S, Stecula A, Thillaisundaram A, Tong C, Yakneen S, Zhong ED, Zielinski M, Zidek A, Bapst V, Kohli P, Jaderberg M, Hassabis D, Jumper JM. Accurate structure prediction of biomolecular interactions with AlphaFold 3. Nature. 2024; 630: 493-500.

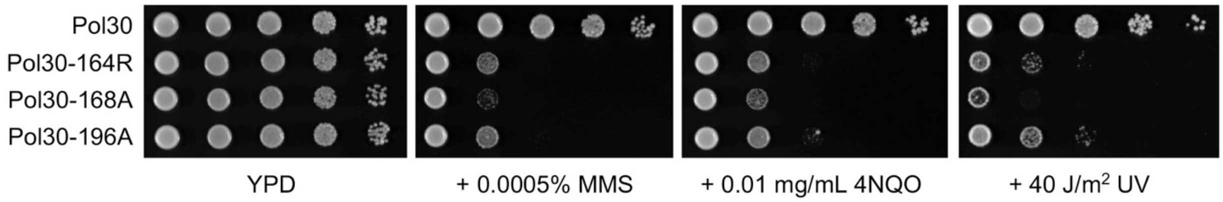
Pol30
Pol30-164R
Pol30-168A
Pol30-196A
YPD
+ 0.0005% MMS
+ 0.01 mg/mL 4NQO
+ 40 J/m2 UV

Figure S1

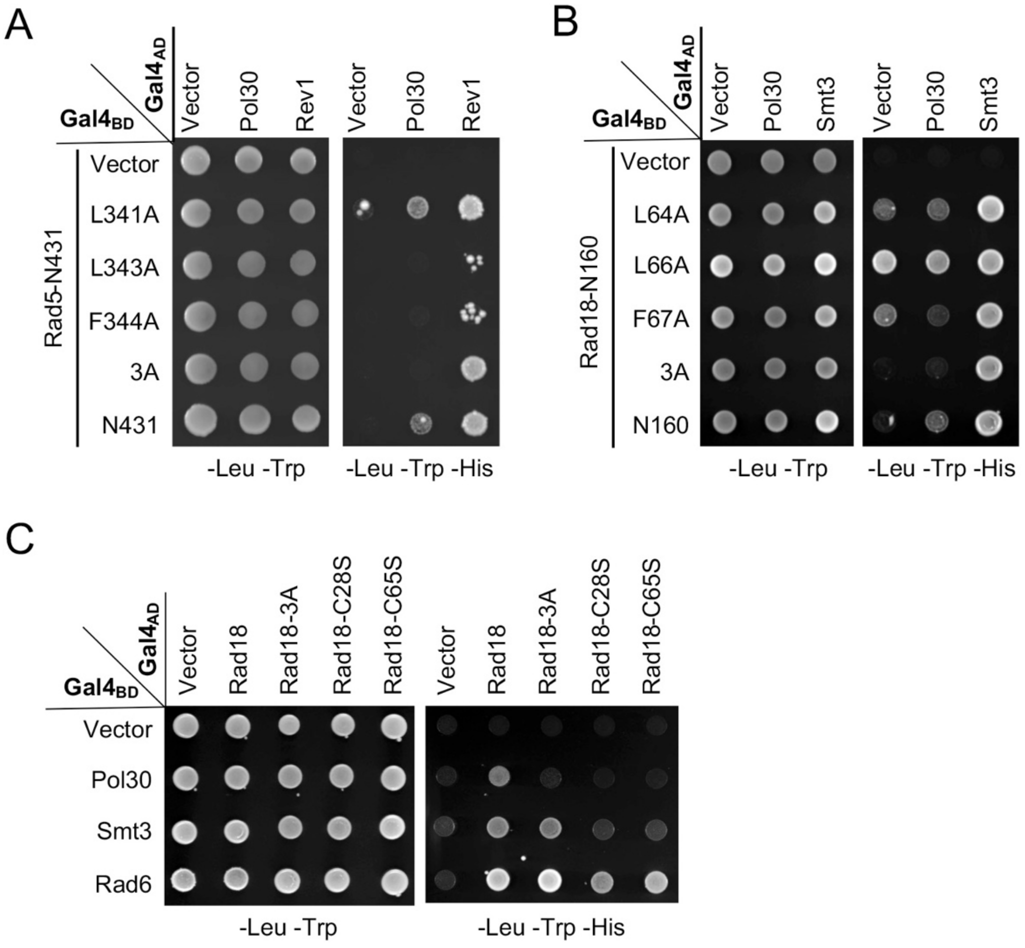
A
Gal4AD
Gal4BD
Vector
Pol30
Rev1
Vector
Pol30
Rev1
Rad5-N431
Vector
L341A
L343A
F344A
3A
N431
-Leu -Trp
-Leu -Trp -His
B
Gal4AD
Gal4BD
Vector
Pol30
Smt3
Vector
Pol30
Smt3
Rad18-N160
Vector
L64A
L66A
F67A
3A
N160
-Leu -Trp
-Leu -Trp -His
C
Gal4AD
Gal4BD
Vector
Rad18
Rad18-3A
Rad18-C28S
Rad18-C65S
Vector
Rad18
Rad18-3A
Rad18-C28S
Rad18-C65S
Vector
Pol30
Smt3
Rad6
-Leu -Trp
-Leu -Trp -His

Figure S2

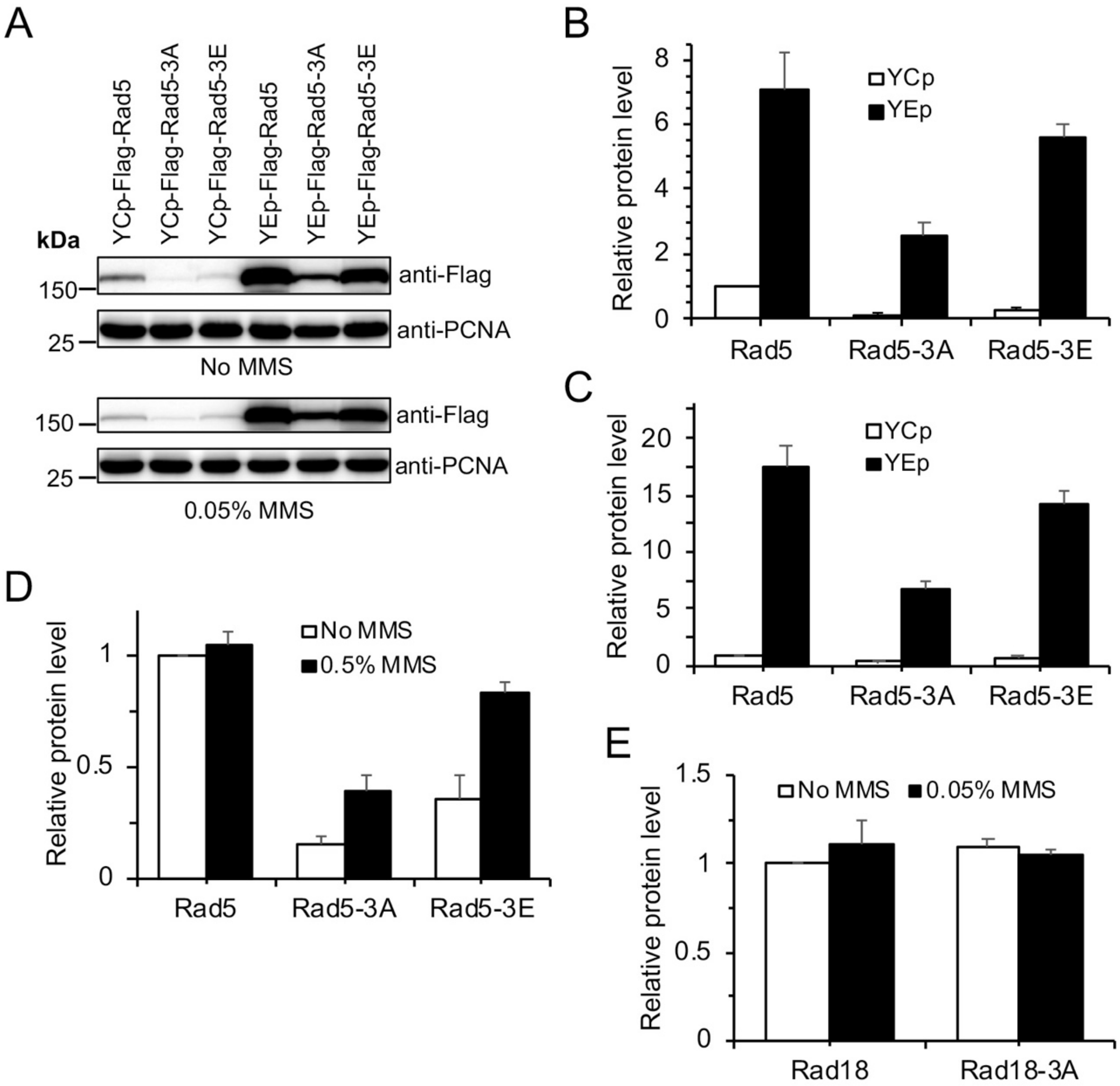
A
kDa
YCp-Flag-Rad5
YCp-Flag-Rad5-3A
YCp-Flag-Rad5-3E
YEp-Flag-Rad5
YEp-Flag-Rad5-3A
YEp-Flag-Rad5-3E
150
anti-Flag
25
anti-PCNA
No MMS
150
anti-Flag
25
anti-PCNA
0.05% MMS
B
Relative protein level
YCp
YEp
0
2
4
6
8
Rad5
Rad5-3A
Rad5-3E
C
Relative protein level
YCp
YEp
0
5
10
15
20
Rad5
Rad5-3A
Rad5-3E
D
Relative protein level
No MMS
0.5% MMS
0
0.5
1
Rad5
Rad5-3A
Rad5-3E
E
Relative protein level
No MMS
0.05% MMS
0
0.5
1
1.5
Rad18
Rad18-3A

Figure S3

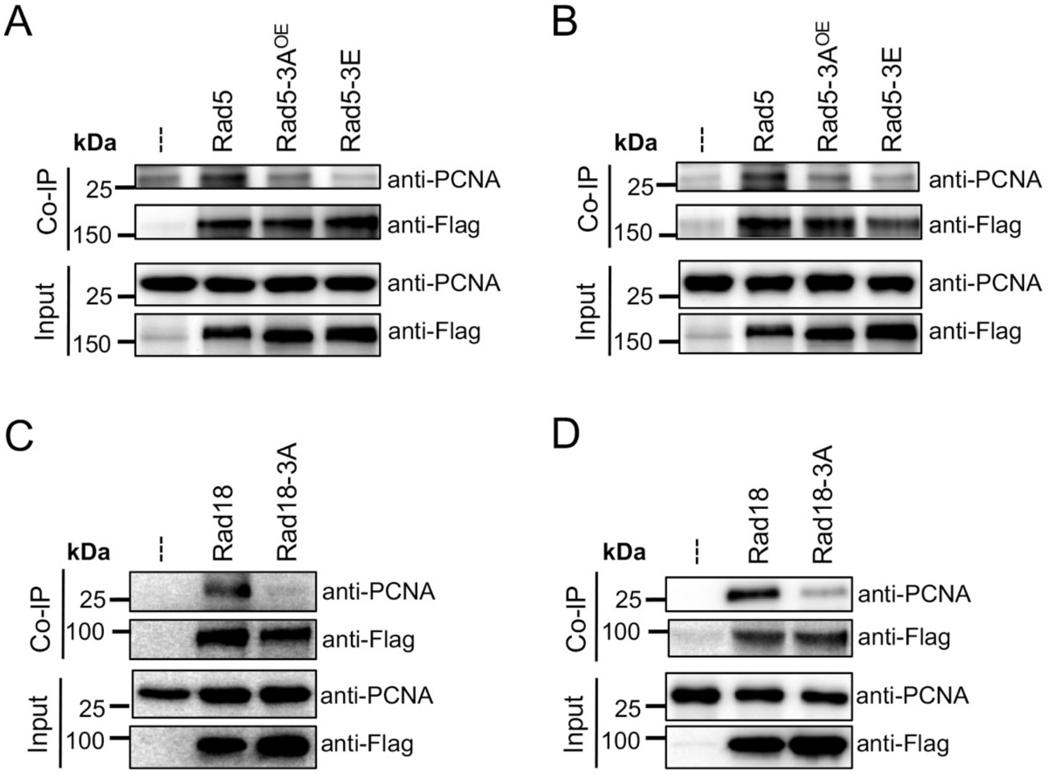
A
kDa
-
Rad5
Rad5-3A^OE
Rad5-3E
Co-IP
Input
25
150
anti-PCNA
anti-Flag
B
C
Rad18
Rad18-3A
100
D

Figure S4

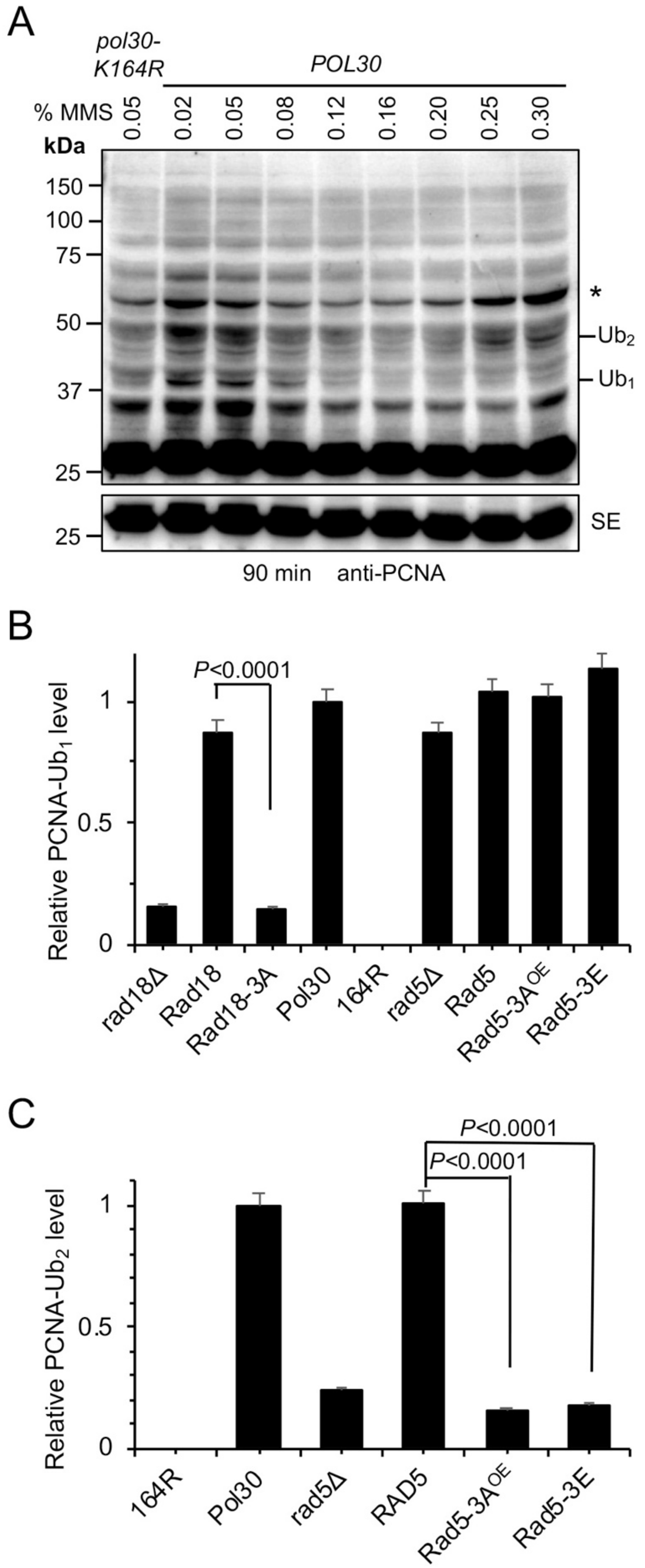
A
pol30-K164R
POL30
% MMS
0.05
0.02
0.05
0.08
0.12
0.16
0.20
0.25
0.30
kDa
150
100
75
50
37
25
*
Ub2
Ub1
SE
25
90 min anti-PCNA
B
Relative PCNA-Ub1 level
P<0.0001
1
0.5
0
rad18Δ
Rad18
Rad18-3A
Pol30
164R
rad5Δ
Rad5
Rad5-3AOE
Rad5-3E
C
Relative PCNA-Ub2 level
P<0.0001
P<0.0001
1
0.5
0
164R
Pol30
rad5Δ
RAD5
Rad5-3AOE
Rad5-3E

Figure S5

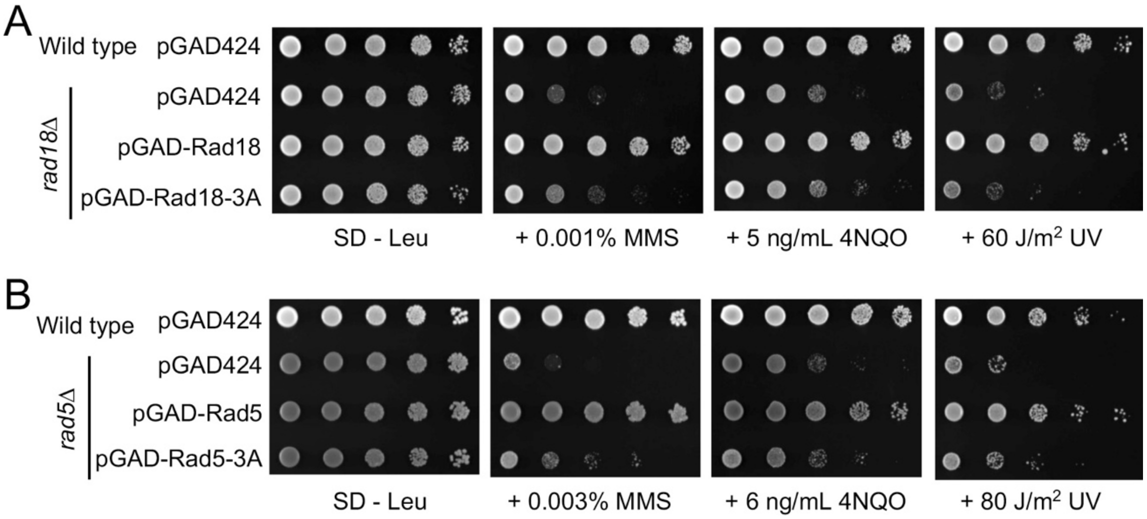
A
Wild type pGAD424
rad18Δ
pGAD424
pGAD-Rad18
pGAD-Rad18-3A
SD - Leu
+ 0.001% MMS
+ 5 ng/mL 4NQO
+ 60 J/m2 UV
B
Wild type pGAD424
rad5Δ
pGAD424
pGAD-Rad5
pGAD-Rad5-3A
SD - Leu
+ 0.003% MMS
+ 6 ng/mL 4NQO
+ 80 J/m2 UV

Figure S6

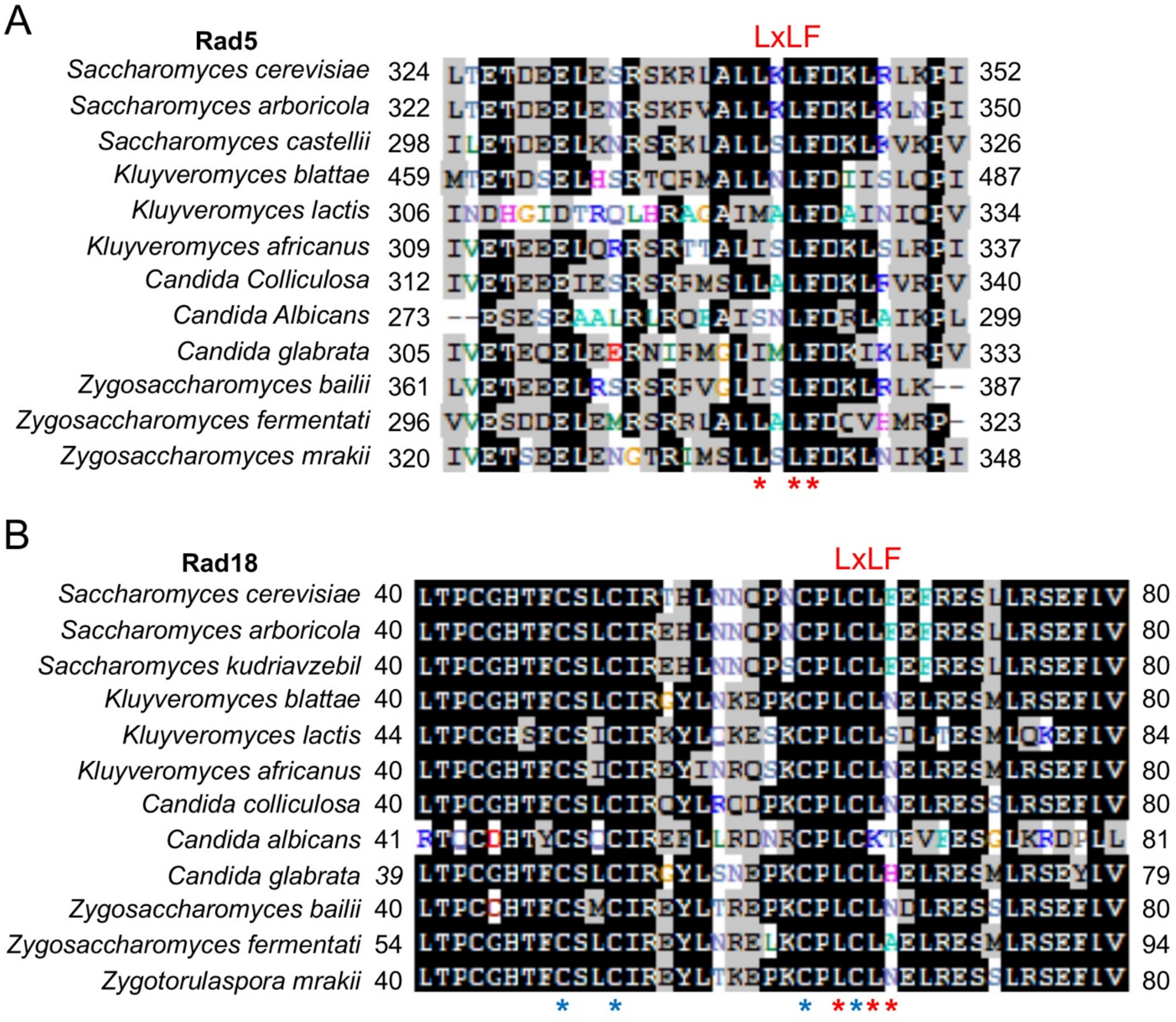
A
Rad5
LxLF
Saccharomyces cerevisiae
Saccharomyces arboricola
Saccharomyces castellii
Kluyveromyces blattae
Kluyveromyces lactis
Kluyveromyces africanus
Candida Colliculosa
Candida Albicans
Candida glabrata
Zygosaccharomyces bailii
Zygosaccharomyces fermentati
Zygosaccharomyces mrakii
B
Rad18
LxLF
Saccharomyces cerevisiae
Saccharomyces arboricola
Saccharomyces kudriavzebil
Kluyveromyces blattae
Kluyveromyces lactis
Kluyveromyces africanus
Candida colliculosa
Candida albicans
Candida glabrata
Zygosaccharomyces bailii
Zygosaccharomyces fermentati
Zygotorulaspora mrakii

Figure S7

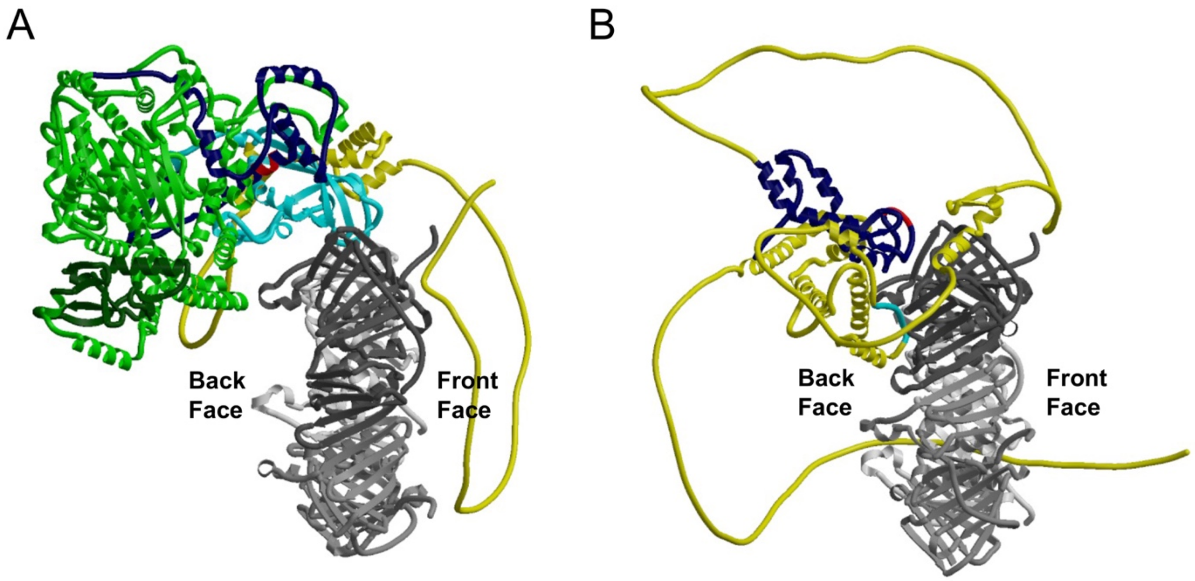

Figure S8
